# Effects of Immersive Virtual Reality Interventions on Symptom Management in Patients With Gastrointestinal Cancer: Systematic Review and Meta-Analysis of Randomized Controlled Trials

**DOI:** 10.2196/86808

**Published:** 2026-07-02

**Authors:** Fangping Chen, Xingyue Guo, Ran Wang, Yan Li, Didi Xu, Yongzhen Wang, Wei Kong, Yin Chen, Luyang Jin, Xuemei Xian

**Affiliations:** 1 Nursing Department, Sir Run Run Shaw Hospital, Zhejiang University School of Medicine, 3 Qingchun East Road, Hangzhou, Zhejiang, 3610016, China, 86 13867406749, 86 0571-86044817; 2 Nursing Department, Hangzhou Hospital of Traditional Chinese Medicine, Hangzhou, Zhejiang, China

**Keywords:** gastrointestinal cancer, virtual reality, systematic review, meta-analysis, nonpharmacological adjunct, symptom management

## Abstract

**Background:**

Patients with gastrointestinal cancers experience a broad range of symptoms, including anxiety, pain, and reduced quality of life. Although immersive virtual reality (IVR) has emerged as a potential intervention, its efficacy specifically in patients with gastrointestinal cancer remains unclear.

**Objective:**

This systematic review and meta-analysis of randomized controlled trials (RCTs) evaluated the effects of IVR on symptom management in patients with gastrointestinal cancer.

**Methods:**

Twelve databases and 1 gray literature source were searched from inception to April 30, 2026. RCTs comparing IVR interventions to routine care or nonimmersive alternatives for symptom management in adults (≥18 years) with gastrointestinal cancer were eligible. Two reviewers independently screened records, extracted data, and assessed risk of bias using the Cochrane RoB 2 tool. The evidence certainty was evaluated using the Grading of Recommendations, Assessment, Development, and Evaluation (GRADE) approach. Random-effects meta-analyses were performed for primary outcomes (anxiety, pain, quality of life) and secondary outcomes (knowledge, length of stay, vital signs, safety). Heterogeneity was explored using subgroup analyses and meta-regression.

**Results:**

Fourteen RCTs were included, comprising individuals (N=837) with colorectal, liver, esophageal, gastric, pancreatic, and biliary tract cancers. IVR interventions—including immersive scenes, interactive games, anatomical models, and cognitive behavioral modules—were primarily delivered during perioperative and chemotherapy periods. Meta-analysis showed that IVR significantly reduced anxiety (standardized mean difference [SMD] −0.58, 95% CI −0.95 to −0.20; *P*=.01; 95% prediction interval [PI] −1.36 to 0.21) and pain (SMD −0.75, 95% CI −1.48 to −0.03; *P*=.04; 95% PI −2.21 to 0.71). Subgroup analysis revealed that the anxiolytic effect was more pronounced when IVR was administered during active treatment and when single sessions lasted ≥20 minutes. Hospital stay was significantly shorter in the IVR group (mean difference −4.11 days, 95% CI −7.39 to −0.82; *P*=.03; 95% PI −13.82 to 5.60 days). No significant effects were detected for quality of life, knowledge acquisition, or vital signs. The evidence certainty was moderate to very low, with common limitations including risk of bias and imprecision.

**Conclusions:**

This meta-analysis provides evidence that IVR is an effective nonpharmacological adjunct for symptom management in patients with gastrointestinal cancer, significantly reducing anxiety and pain when implemented during active treatment for at least 20 minutes. However, these findings should be interpreted with caution due to moderate to high heterogeneity, substantial risk of bias in the included studies, and low to very low GRADE evidence certainty. While the 95% CIs indicate a statistically significant average effect, the wide 95% PIs suggest that the true effect in future clinical settings may vary considerably, ranging from marked benefit to negligible impact. These results support the integration of IVR into perioperative and chemotherapy care pathways while underscoring the need for larger, more rigorously designed trials to establish definitive conclusions.

## Introduction

Gastrointestinal cancers represent a significant global health burden, accounting for 24% of cancer-related morbidity and 33.3% of mortality worldwide [1]. Clinical management usually uses multimodal integrated strategies encompassing surgical resection, chemotherapy, and radiotherapy, often constituting a protracted and complex process [2]. Throughout this journey, patients frequently experience an interplay of physical pain and psychological distress [3]. Approximately 53% of patients with gastrointestinal cancer report moderate to severe pain [4]. This pain is predominantly visceral in nature, characterized by vague localization and heightened sensitivity to emotional states [5]. Concurrently, 56% of patients experience clinically significant anxiety, which often peaks prior to treatment [4].

Evidence suggests a bidirectional exacerbation mechanism between pain and anxiety. Anxiety activates the hypothalamic-pituitary-adrenal axis and the sympathetic nervous system [6], thereby lowering pain thresholds and amplifying pain perception [7]. In turn, persistent pain intensifies feelings of helplessness and fear, further worsening anxiety [8]. This vicious cycle of co-occurring physical and psychological symptoms not only diminishes overall quality of life but may also trigger a cascade of adverse consequences. Specifically, heightened psychophysiological stress can impair patients’ ability to absorb disease- and treatment-related knowledge, exacerbate preoperative stress responses, cause fluctuations in vital signs during and after procedures, prolong hospital stay, and increase the risk of complications [9,10]. Therefore, interventions capable of simultaneously modulating both physical and psychological states are urgently needed. Such approaches hold considerable promise for improving symptom experience, accelerating recovery, and optimizing clinical outcomes in patients with gastrointestinal cancer.

Several interventions have been developed to support symptom management in this population, including aerobic exercise, relaxation training, and music therapy [11-13]. However, most of these strategies target a single domain. For instance, aerobic exercise primarily addresses physical conditioning [11], whereas relaxation training focuses on alleviating psychological distress [12]. In routine clinical practice, integrated interventions that effectively address both physical and psychological dimensions remain limited [14]. In recent years, immersive virtual reality (IVR) has emerged as a novel nonpharmacological intervention and has been gradually adopted across various medical fields [15]. In gastrointestinal cancer care, IVR has been applied at multiple critical junctures. Preoperatively, IVR can use anatomical models and simulated scenarios to help patients visualize surgical procedures and reduce fear of the unknown [16]. Intraoperatively, immersive natural scenes or interactive games can divert attention and alleviate tension and pain during procedures performed under local anesthesia [17]. Postoperatively, virtual reality (VR)–based games can facilitate early mobilization and rehabilitation [18,19]. Additionally, VR-delivered relaxation environments have been used to ameliorate anxiety [20]. The core strength of IVR lies in its capacity to generate a high degree of immersion and sense of presence. By simultaneously engaging multiple sensory channels, IVR can effectively distract patients to reduce pain perception while also activating the parasympathetic nervous system through immersive relaxation, thereby mitigating anxiety [21,22]. This capability for synergistic psychophysiological regulation positions IVR as a potentially powerful tool for symptom management.

Preliminary studies have reported positive effects of IVR in reducing anxiety and pain, improving quality of life, and shortening hospital stay among patients with gastrointestinal cancer [15,18]. However, existing systematic reviews are limited by several factors. Many include heterogeneous cancer populations without stratification by tumor type [19], exhibit substantial variability in intervention protocols [15], and lack in-depth analysis of key moderating factors such as intervention timing and session duration [18]. To date, no high-quality systematic review has specifically focused on the gastrointestinal cancer population to systematically evaluate the effects of IVR on symptom management and to explore factors that may influence these effects.

Therefore, this systematic review and meta-analysis aims to comprehensively evaluate the effectiveness of IVR interventions for symptom management in patients with gastrointestinal cancer. Outcomes assessed included anxiety, pain, and other indicators relevant to symptom management. Furthermore, subgroup analyses and meta-regression were conducted to explore the potential moderating effects of intervention characteristics on IVR efficacy. The findings are intended to provide evidence-based guidance for the precise implementation of IVR in clinical practice and to inform the design of future high-quality research.

## Methods

### Design

The meta-analysis was conducted according to the PRISMA (Preferred Reporting Items for Systematic Reviews and Meta-Analyses) 2020 statement [23]. The completed PRISMA 2020 checklists for the main manuscript (Checklist 1) and the abstract (Checklist 2) are provided. The study protocol was registered in the PROSPERO database (CRD420251113000).

### Ethical Considerations

Ethics approval was not required for this systematic review because it did not involve the recruitment of patients.

### Search Strategy

The search strategy was developed de novo and was not adapted from prior reviews. It was reported in accordance with the PRISMA-S (Preferred Reporting Items for Systematic reviews and Meta-Analyses literature search extension) guideline [24]. The PRISMA-S checklist is provided as Checklist 3.

We systematically searched 12 databases and 1 gray literature source, including PubMed (NLM); Web of Science (Clarivate Analytics); Institute of Electrical and Electronics Engineers (IEEE) Xplore; Scopus (Elsevier); CINAHL (EBSCOhost); PsycINFO (EBSCOhost); EMBASE (Elsevier); Cochrane Central Register of Controlled Trials (CENTRAL; Cochrane Library); and Chinese databases, encompassing China National Knowledge Infrastructure (CNKI), Wanfang Chinese Database, China Science and Technology Journal Database (VIP), and China Biology Medicine disc (CBM). No simultaneous multidatabase searches were performed on any platform, and each database was searched separately. The CENTRAL database was searched as a study registry for ongoing and completed randomized controlled trials (RCTs). All databases were initially searched from inception to August 15, 2025. An updated search was conducted on April 30, 2026, to identify any additional eligible studies published in the interim. The updated search identified 2 additional eligible studies, which were incorporated into the final synthesis. In addition to database searches, gray literature was searched via ProQuest Dissertations and Theses Global (ProQuest), and the reference lists of all included studies were manually screened to identify any other eligible studies. No online resources or print sources were browsed.

The search strategy was developed by FC in consultation with XG, using a combination of MeSH terms and free-text keywords, related to gastrointestinal cancer (eg, gastric/stomach, colorectal/colon/rectal), IVR (eg, virtual reality and head-mounted display), and RCTs. The search strategy was peer-reviewed by XX using the PRESS (Peer Review of Electronic Search Strategies) checklist [25] prior to execution. The search strategy was first developed in PubMed and subsequently adapted for each database, accounting for differences in syntax, controlled vocabulary, and search operators across platforms. No database-level date or language limits were applied during the searches. Language eligibility was assessed during the screening phase in accordance with the inclusion and exclusion criteria. The full search strategies for all databases are provided in Multimedia Appendix 1.

### Inclusion and Exclusion Criteria

Inclusion criteria were formulated based on the PICOS framework:

1. Population (P): patients aged 18 years or older with gastrointestinal cancer—including esophageal cancer, gastric cancer, pancreatic cancer, liver cancer, rectal cancer, colon cancer, and biliary tract cancer, according to the World Health Organization classification of digestive system cancers [26]—were included.
2. Intervention (I): IVR technology was used for intervention, encompassing virtual telehealth, virtual environment, VR anatomical model, virtual interactive game, etc [27].
3. Comparison (C): routine care or non-IVR technology were used for intervention.
4. Outcomes (O): the primary outcomes were anxiety and pain, which represent the core physical and psychological symptoms. Secondary outcomes included other indicators related to symptom management, including knowledge, vital signs (heart rate and blood pressure), length of hospital stay, quality of life, and safety.
5. Study design (S): RCTs were included.

The exclusion criteria were as follows: (1) studies for which the full text could not be obtained or that were conference abstracts, editorials, or protocols and (2) studies not published in Chinese or English.

### Study Selection and Data Extraction

All search results were imported into NoteExpress (version 4.1.0.10021; Beijing Aegean Software) for data management. After all duplicate articles were removed, 2 researchers (FC and XG) independently conducted a primary screening by examining the titles and abstracts of the studies. Then, they reviewed the full text of each paper according to the inclusion and exclusion criteria and performed data extraction independently, including author, study year, country, study design, sample size, participants (age and sex), time point, intervention types, intervention setting, provider, intervention methods, intervention content, intervention duration, control conditions, outcomes, and instruments. The data extraction form was developed and pilot-tested based on the Cochrane Handbook for Systematic Reviews of Interventions [28]. Meanwhile, they cross-checked the screening results. Any discrepancies were resolved by discussion or, if necessary, inclusion of a third researcher (XX). In the interest of transparency, the PRISMA checklist has been included as Checklist 1. This checklist details our adherence to reporting standards, facilitating the assessment and reproducibility of our research.

### Assessment of Risk of Bias and Certainty of Evidence

The risk of bias was assessed by 2 researchers (FC and XG) independently according to the Revised Cochrane risk-of-bias tool for randomized trials (RoB 2) [29]. Disagreements were resolved either by consensus or by a third partner (XX). Five domains were considered in the evaluation process: randomization process, deviations from intended interventions, missing outcome data, measurement of the outcome, and selection of the reported result. Each domain was assessed for risk of bias, and the overall bias of studies was then identified to be “low risk,” “some concerns,” and “high risk.”

The Grading of Recommendations, Assessment, Development, and Evaluation (GRADE) approach was used by 2 independent researchers (RW and YL) to assess the certainty of the evidence. The GRADE approach is a widely recognized tool for grading evidence certainty in systematic reviews and clinical practice guidelines. It categorizes evidence certainty into 4 levels, “very low,” “low,” “moderate,” or “high,” each determined by specific criteria [30]. Factors that may diminish evidence certainty (eg, risk of bias, inconsistency, indirectness, imprecision, and publication bias) as well as those that may enhance it (eg, large effect, plausible confounding, and dose-response) were evaluated [31]. In the event of discrepancies in the certainty ratings among the reviewers, a third reviewer (XX) was consulted.

### Data Synthesis and Statistical Analysis

For continuous variables, with the exception of vital signs where most studies reported the mean difference (MD) and SD, all other indicators were expressed as mean (SD) post intervention. For studies that measured outcomes at multiple time points, the first postintervention measurement was selected for the meta-analysis of the main effects. Furthermore, we calculated the MD or standardized mean difference (SMD) with the corresponding 95% CI, based on whether the outcomes were measured by the same scales or not. For categorical variables, we calculated the risk ratio with corresponding 95% CI. Statistical significance was determined using a 2-sided *P* value threshold of <.05. The effect sizes were classified as small (0.20‐0.49), moderate (0.50‐0.79), or large (≥0.80) [32]. If the study did not provide mean or SD, we converted available data into mean (SD), median (95% CI), or median (IQR), according to the Cochrane Handbook for Systematic Reviews of Interventions [28].

Where outcome data were incompletely reported in included studies (eg, missing SDs or only median reported without dispersion measures), we contacted the corresponding authors via email to request the missing information. A maximum of 2 email attempts were made, spaced 2 weeks apart. For studies where authors did not respond or data remained unavailable, the study was included in the systematic review narrative but excluded from the specific quantitative meta-analysis for that outcome. This approach was documented in the study characteristics table and noted as a limitation.

To address the anticipated heterogeneity arising from variations in clinical settings and populations across studies, random-effects models were preferred to the usual statistical tests [33]. In addition, the Hartung-Knapp-Sidik-Jonkman method was used to reduce the production of false positives inherent to the DerSimonian-Laird method and to obtain more robust estimates of variance [34]. Heterogeneity was quantified using the τ^2^ statistic [35], while the *I*^2^ statistic was reported for descriptive reference (with values of 25%, 50%, and 75% commonly indicating low, moderate, and high levels, respectively) [36]. To better assess the practical implications of heterogeneity, a 95% prediction interval was also calculated [37] to estimate the range within which the true effect of a future, similar study would be expected to fall, thereby providing a more clinically meaningful interpretation of heterogeneity. If meta-analyses showed high heterogeneity, we performed subgroup analyses and meta-regression to look into any potential study-level factor influencing the treatment effect. We conducted sensitivity analyses on the outcome measures using the leave-one-out method to assess the robustness of the combined results.

## Results

### Study Selection

**Figure 1.**
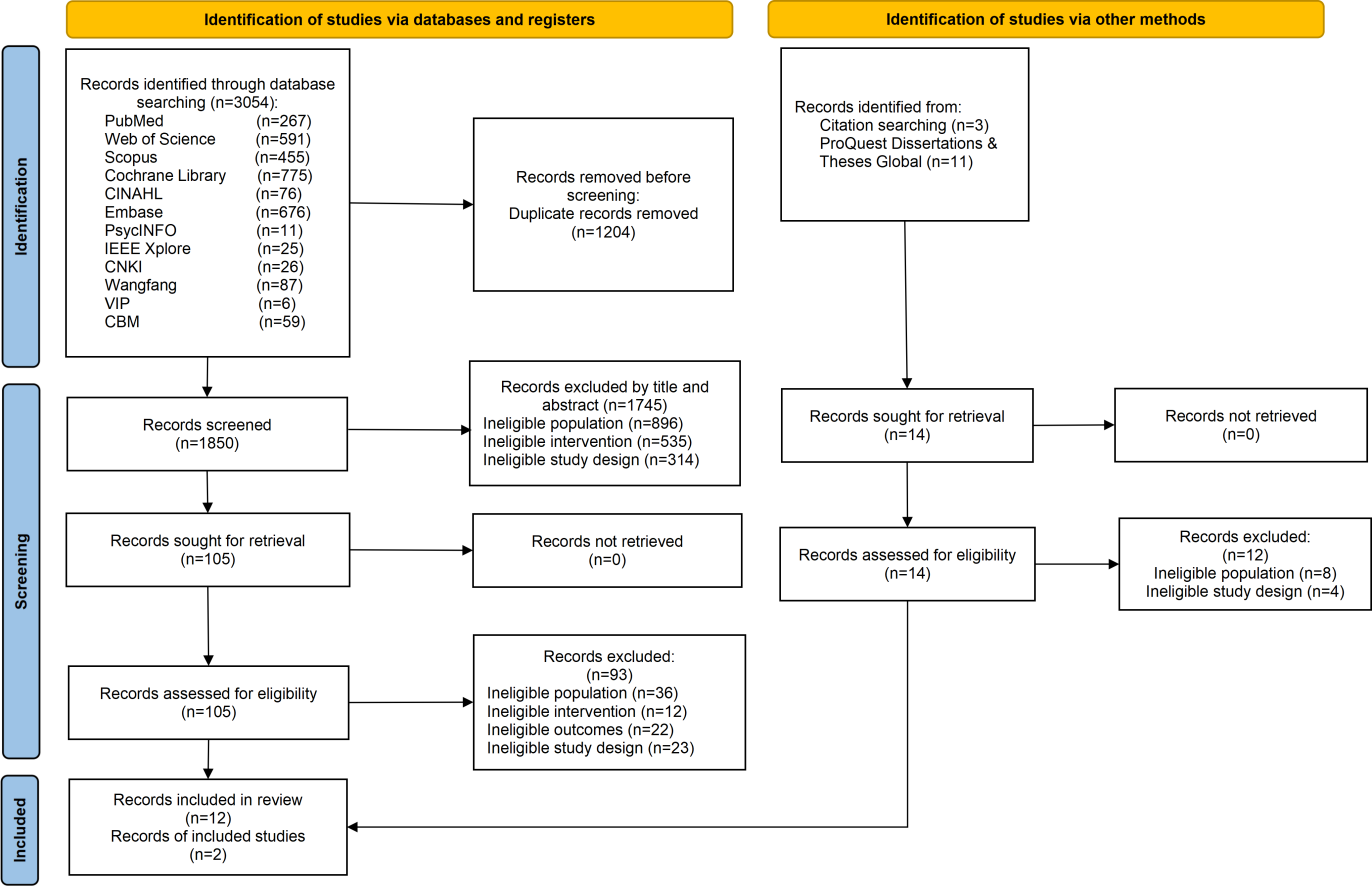
PRISMA (preferred reporting items for systematic reviews and meta-analyses) flowchart. CBM: China Biology Medicine disc; CNKI: China National Knowledge Infrastructure; IEEE, Institute of Electrical and Electronics Engineers; VIP: China Science and Technology Journal Database; Wangfang: Wanfang Chinese Database.

A total of 3054 records were identified through database searches, with an additional 14 records retrieved via other methods, including citation searching and ProQuest Dissertations & Theses Global. After removing 1204 duplicates, 1850 articles underwent title and abstract screening, of which 1745 were excluded as irrelevant. Full texts of 105 articles were retrieved for eligibility assessment, and 93 were excluded due to ineligible populations (n=36), intervention (n=12), outcomes (n=22), and study design (n=23). Of the 14 records identified through other methods, 12 were excluded owing to ineligible population (n=8) or ineligible study design (n=4). Ultimately, 14 studies were included in this review (see Figure 1).

### Study Characteristics

The characteristics of the included studies are summarized in Table 1. Among the 14 included RCTs, 78.6% (n=11) of the studies enrolled older patients with an average age of ≥60 years [38]. Twelve studies were 2-arm parallel RCTs [11,12,16,17,20,39-45], one study used a 3-arm design [13], and another used a 4-arm design [46]. Geographically, studies were conducted between 2020 and 2026, including China (n=6) [11,17,20,39-41], Germany (n=2) [13,43], South Korea (n=2) [16,45], Japan (n=1) [12], Spain (n=1) [44], Australia (n=1) [42], and England (n=1) [46]. A total of 837 patients were included across all studies, with sample sizes ranging from 9 to 126 participants, encompassing individuals with colorectal cancer (n=7) [11,13,20,42-44,46], liver cancer (n=3) [16,17,41] and esophageal cancer (n=1) [39]. Additionally, 3 studies included mixed populations of patients with gastrointestinal cancer, including colorectal, esophageal, gastric, pancreatic, biliary tract, and liver cancers [12,40,45].

**Table 1.** Study characteristics.

Study	Country	Study design	Participants (sample size)	Age in years, mean (SD)		Women, n (%)		Time point	Intervention group						Control group	Outcomes (instruments)
				IG^a^	CG^b^	IG	CG		Type	Setting	Provider	Method	Content	Duration		
Kamada et al [12], 2025	Japan	Two-arm RCT^c^	Colorectal, esophageal, gastric, and pancreatic cancers (N=10; IG: n=5, CG: n=5)	65 (8.1)	61.2 (13.4)	2 (40)	3 (60)	Intraoperative	Distraction technique	Operating room	Physician	VR^d^ immersive scenes (space/forest/ocean)	Immerse patients through nature visuals, sounds, and lyrics, guiding their breathing rhythm to promote relaxation	Once during procedure; 22.7-41.5 min	Standard therapy	Anxiety (VAS)^e^; pain (NRS)^f^
Kim et al [45], 2026	South Korea	Two-arm RCT	Pancreatic, biliary tract, and liver cancers (N=45; IG: n=22, CG: n=23)	67.5 (7.1)	68.4 (8.1)	7 (31.8)	10 (43.5)	Postoperative	Distraction technique	Ward	Physician	VR immersive nature scenes	Immerse patients through nature visuals, calming sounds, guiding their breathing rhythm to promote relaxation	Twice (30 min and 4 h postoperatively); 7 min/session	VR without guided breathing	Pain (NRS); safety
Li et al [20], 2026	China	Two-arm RCT	Colorectal cancer (N=84; IG: n=42, CG: n=42)	46.21 (9)	46.48 (9.7)	18 (42.9)	20 (47.6)	Perioperative	Cognitive behavioral therapy (CBT)	Not restricted	Nurse	Self-led VR-CBT^g^ with immersive scenes (beach/nature) and interactive virtual nurse delivering CBT techniques	Cognitive reconstruction (identify/modify maladaptive beliefs) and behavioral techniques (breath/muscle relaxation, problem-solving)	Seven sessions over 8 weeks; 30 min/session	WeChat health consultations and usual care	Anxiety (HADS)^h^
Schrempf et al [13], 2022	Germany	Three-arm RCT	Colorectal cancer (N=36; IG: n=18, CG: n=18)	55.8 (10.2)	63.4 (8.2)	8 (44.4)	4 (22.2)	Preoperative	Distraction technique	Ward	Physician	VR immersive scenes; VR interactive games	Mindfulness meditation, interactive breathing exercises	Twice daily; 7-10 min/session	Music therapy	Quality of life (EORTC QLQ-C30^i^, EORTC-SF2)^j^; length of hospital stay (days); BP^k^; HR^l^
Schrempf et al [43], 2023	Germany	Two-arm RCT	Colorectal cancer (N=62; IG: n=31, CG: n=31)	60.4 (9.5)	60.9 (9.7)	12 (38.7)	13 (41.9)	Postoperative	Rehabilitation training	Ward	Physician	VR immersive scenes; VR interactive games	Promote active movement through the completion of in-game tasks (rowing/cycling)	Daily; 30 min; from postoperative day 1 to discharge	Standard rehabilitation therapy	Quality of life (EQ-5D-5L); length of hospital stay (days); safety
Shepherd et al [42], 2024	Australia	Two-arm RCT	Colorectal cancer (N=9; IG: n=5, CG: n=4)	68 (8.8)	54 (6.2)	2 (40)	3 (75)	Preoperative	Exposure therapy	Outpatient department	Physician	VR immersive scenes,VR anatomical models	Preoperative education using 3D VR models of colon and patient's own anatomy	Once preoperatively; 21-45.53 min	Conventional preopperative education	Knowledge (self-developed); safety
Song et al [40], 2020	China	Two-arm RCT	Colorectal, esophageal, and gastric cancers (N=64; IG: n=32, CG: n=32)	64.4	61.2	9 (28.1)	11 (34.4)	Preoperative	Distraction technique	Ward	Physician	VR immersive scenes (green/blue)	Experience different landscapes	3-5 sessions/week; 5 min 20 s/session	VR grayscale scenes	Pain (self-developed); HR; BP
Turrado et al [44], 2021	Spain	Two-arm RCT	Colorectal cancer (N=126; IG: n=58, CG: n=68)	63.3 (33.4)	68 (27.2)	25 (43.1)	28 (41.2)	Preoperative	Exposure therapy	Ward	Physician	VR immersive scenes; VR videos	Experience the surgical procedure	16 min 34 s; unlimited sessions	Usual care	Anxiety (STAI)^m^; length of hospital stay (days)
Xing et al [41], 2023	China	Two-arm RCT	Liver cancer (N=74; IG: n=37, CG: n=37)	59.5 (11.5)	58.8 (12.2)	5 (13.5)	4 (10.8)	Intraoperative	Distraction technique	Operating room	Physician	VR immersive scenes (nature/hypnosis/breath)	Immerse relaxation scenes	Once during TACE^n^; >60 min	Usual care	Anxiety (STAI); pain (NRS); BP; HR
Xue et al [17], 2024	China	Two-arm RCT	Liver cancer (N=76; IG: n=38, CG: n=38)	60.3 (8.4)	61.5 (10.4)	9 (23.7)	8 (21.1)	Intraoperative	Distraction technique	Operating room	Physician	VR immersive scenes (nature/mindfulness/music)	Immersive relaxation scenes	Once during TACE; 43.8-70.5 min	Usual education	Anxiety (SAS)^o^; pain (NRS); safety (NCI-CTCAE^p^ 5.0); BP; HR
Yang et al [16], 2024	South Korea	Two-arm RCT	Liver cancer (N=88; IG: n=44, CG: n=44)	58.1 (8)	59.7 (7.3)	12 (27.3)	10 (22.7)	Preoperative	Education	Ward	Physician	VR immersive scenes; VR videos; VR anatomical models	Interactive liver anatomy education	Once; 8 min 34 s	Usual education	Knowledge (self-developed); anxiety (STAI and VAS)
Yiasemidou et al [46], 2021	England	Four-arm RCT	Colorectal cancer (N=49; IG: n=12, CG: n=12)	61.7 (34.1)	71.6 (17.8)	Not mentioned	Not mentioned	Preoperative	Exposure therapy	Ward	Physician	VR anatomical models	Mental rehearsal with VR anatomical model	Once preoperatively; 20-30 min	Usual education	Length of hospital stay (days)
Yue et al [11], 2025	China	Two-arm RCT	Colorectal cancer (N=70; IG: n=35, CG: n=35)	64.3 (6.5)	64.5 (10.3)	11 (31.4)	12 (34.3)	Prechemotherapy	Distraction technique	VR experience room	Nurse	VR immersive scenes (forest/beach)	Experience immersive environments, promote relaxation and stress relief	15 min/day; ≥5 sessions/cycle; 6 cycles	Usual care	Anxiety (HADS); quality of life (EORTC QLQ-C30)
Zhao et al [39], 2024	China	Two-arm RCT	Esophageal cancer (N=44; IG: n=22, CG: n=22)	67.7 (5.3)	67.1 (5)	3 (13.6)	5 (22.7)	Intrachemotherapy	Distraction technique	Ward	Nurse	VR immersive scenes	Experience nature scenes and fishing interaction	Seven sessions; 10-45 min/session	Usual care	Pain (BPI)^q^; quality of life (EORTC QLQ-C30)

a IG: intervention group.

b CG: control group.

c RCT: randomized controlled trial.

d VR: virtual reality.

e VAS: Visual Analog Scale.

f NRS: Numerical Rating Scale.

g VR-CBT: virtual reality cognitive behavioral therapy.

h HADS: Hospital Anxiety and Depression Scale.

i EORTC QLQ-C30: European Organisation for Research and Treatment of Cancer Quality of Life Questionnaire Core 30.

j EORTC-SF2: European Organisation for Research and Treatment of Cancer Quality of Life Questionnaire Short Form 2.

k BP: blood pressure.

l HR: heart rate.

m STAI: State-Trait Anxiety Inventory.

n TACE: transarterial chemoembolization.

o SAS: Self-Rating Anxiety Scale.

p NCI-CTCAE: National Cancer Institute Common Terminology Criteria for Adverse Events.

q BPI: Brief Pain Inventory.

IVR is primarily used in perioperative and chemotherapy settings for distraction, exposure therapy, rehabilitation training, knowledge education, and cognitive behavioral therapy. To implement the interventions, 12 studies (85.7%) used VR immersive scenes (eg, nature and ocean) [11-13,16,17,39-45], 2 used VR interactive games [13,43], 3 made use of VR anatomical models [16,42,46], and 2 incorporated VR videos [16,44]. One study delivered a structured VR-based cognitive behavioral therapy program integrating cognitive reconstruction and behavioral techniques [20]. Moreover, 6 of the 14 (42.9%) studies combined 2 or more VR interventions [13,16,20,42-44]. Interventions were administered across diverse clinical settings: 8 of the 14 (57.1%) studies were conducted in hospital wards [13,16,39,40,43-46]; interventions were administered during minor procedures under local anesthesia in operating theaters, such as implantable port placement, in 3 studies [12,17,41]; in an outpatient department in 1 study [42]; in a dedicated VR experience room in 1 study [11]; and without restriction on setting in 1 study [20]. These interventions were delivered by health care providers, with 11 studies (78.6%) involving physicians [12,13,16,17,40-46] and 3 (21.4%) involving nurses [11,20,39]. Interventions were primarily implemented at 3 time points: before treatment (eg, surgery, radiotherapy, and chemotherapy; 7/14, 50%) [11,13,16,40,42,44,46], during treatment (4/14, 28.6%) [12,17,39,41], and after treatment (2/14, 14.3%) [43,45], and 1 study spanned the entire perioperative period from presurgery to 6 weeks post discharge [20]. The duration and frequency of interventions exhibited significant differences, ranging from 7 studies involving single treatment sessions [12,16,17,41,42,45,46] to 7 studies using systematic programs lasting up to 2 months, with multiple weekly sessions [11,13,20,39,40,43,44].

### Risk of Bias in Studies

**Figure 2.**
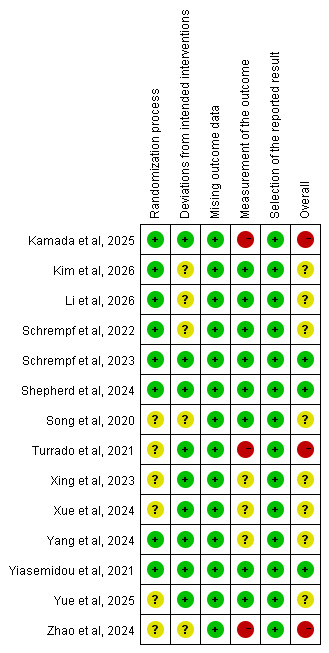
Risk of bias summary [11-13,16,17,20,39-46].

The results of the risk of bias assessment are presented in Figure 2. Among the 14 included studies, 3 (21.4%) demonstrated a high overall risk of bias [12,39,44], 8 (57.1%) were rated as having some concerns [11,13,16,17,20,40,41,45], and 3 (21.4%) studies had a low overall risk of bias [42,43,46]. Six of the 14 (42.9%) studies had some concerns regarding the randomization process (domain 1) because information on allocation concealment was not provided [11,17,39-41,44]. Owing to the nature of the interventions, multiple studies did not specify whether blinding was applied to either the intervention providers or participants. Two studies implemented blinding for staff responsible for patient treatment and care, as well as for those assessing outcomes [20,43]. Another study had the control group view placebo scenes through VR headsets [40]. One study excluded intervention providers from VR usage, with rules being conveyed solely through standard verbal instructions [42]. The participant attrition in 3 studies might raise some concerns about the risk of bias due to potential deviation from the intended intervention (domain 2) [13,40], but 2 of them used an intention-to-treat analysis to address the missing data [13,20]. Additionally, 1 study did not mention whether deviations from the intended intervention occurred due to factors inherent to the experimental environment, raising some concerns about the risk of bias due to potential deviation from the intended intervention (domain 2) [39]. Three studies presented some concern about the risk of bias in outcome measurement (domain 4) because some self-reporting tools were administered by health care professionals [16,17,41]. Three studies presented some concern about the risk of bias in outcome measurement (domain 4) because they used self-report measures, unlike the remaining studies that used objective measures [12,39,44].

### Certainty of Evidence

The overall certainty of evidence for each outcome, evaluated using the GRADE framework, is summarized in Table 2. Evidence of moderate certainty was found for the effects on knowledge, length of stay, systolic blood pressure, and heart rate. Ratings were downgraded primarily due to serious imprecision (wide CIs and/or limited sample sizes). Evidence of low certainty was identified for anxiety, pain, and diastolic blood pressure, with downgrades due to serious risk of bias, serious inconsistency, or serious imprecision. Evidence for quality of life was of very low certainty, reflecting serious concerns in risk of bias, inconsistency, and imprecision.

**Table 2.** Summary of findings and certainty of evidence (Grading of Recommendations, Assessment, Development, and Evaluation [GRADE]).

Certainty assessment							Participants, n		Absolute effect (95% CI)	Certainty
Studies, n	Study design	Risk of bias	Inconsistency	Indirectness	Imprecision	Other considerations	Intervention	Control		
Anxiety										
7	Randomized trials	Serious^a^	Serious^b^	Not serious	Not serious	None	259	269	SMD^c^ 0.58 lower (0.89 lower to 0.27 lower)	⨁⨁◯◯ Low^a^^b^^d^
Pain										
6	Randomized trials	Serious^a^	Serious^b^	Not serious	Not serious	None	156	156	SMD 0.75 lower (1.26 lower to 0.25 lower)	⨁⨁◯◯ Low^a^^b^^d^
Quality of life										
4	Randomized trials	Serious^a^	Serious^b^	Not serious	Serious^d^	None	106	106	SMD 0.83 higher (3.76 lower to 5.43 higher)	⨁◯◯◯ Very low^a^^b^^d^
Knowledge										
2	Randomized trials	Not serious	Not serious	Not serious	Serious^d^^e^	None	49	48	SMD 1.21 higher (0.86 lower to 3.27 higher)	⨁⨁⨁◯ Moderate^e^
Length of stay										
3	Randomized trials	Not serious	Not serious	Not serious	Serious^e^	None	61	61	MD^f^ 4.11 lower (7.39 lower to 0.82 lower)	⨁⨁⨁◯ Moderate^e^
Systolic blood pressure										
4	Randomized trials	Not serious	Not serious	Not serious	Serious^d^	None	125	125	MD 2.44 lower (5.51 lower to 0.64 higher)	⨁⨁⨁◯ Moderate^d^
Diastolic blood pressure										
4	Randomized trials	Not serious	Serious^b^	Not serious	Serious^d^	None	125	125	MD 3.78 lower (9.23 lower to 1.67 higher)	⨁⨁◯◯ Low^b^^d^
Heart rate										
4	Randomized trials	Not serious	Not serious	Not serious	Serious^d^	None	125	125	MD 0.1 lower (0.65 lower to 0.45 higher)	⨁⨁⨁◯ Moderate^d^

a At least 1 study was rated as high risk.

b If the *I*2 statistic exceeds 50% and the source of heterogeneity cannot be explained.

c SMD: standardized mean difference.

d The 95% CI is excessively wide or spans a clinically significant threshold.

e The number of included studies is too small or the sample size is inadequate.

f MD: mean difference.

### Effectiveness of Interventions on Primary Outcome Measures

#### Anxiety

**Figure 3.**
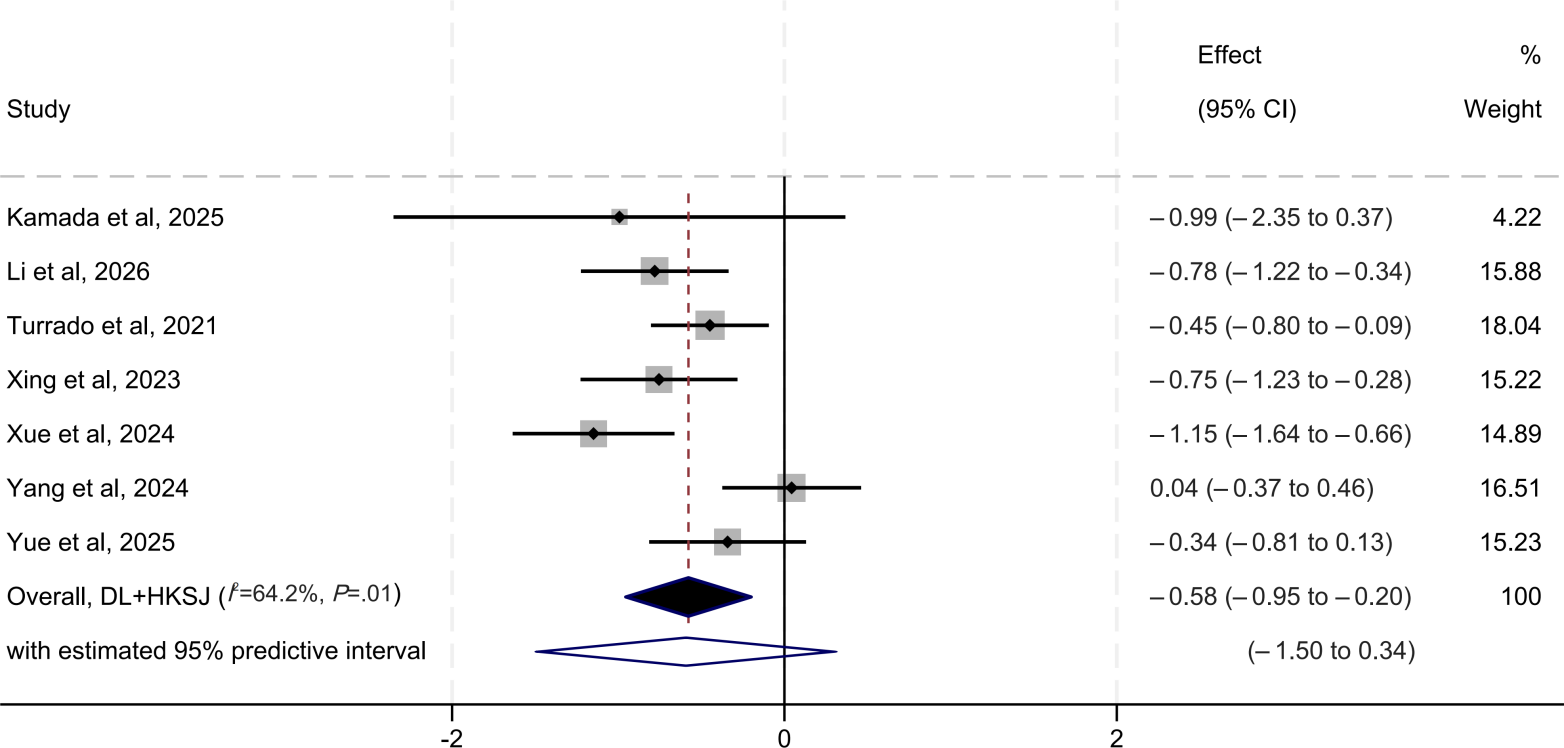
Forest plot of the effect of immersive virtual reality on anxiety [11,12,16,17,20,41,44]. Weights are from a random-effects model. DL+HKSJ: DerSimonian-Laird method with the Hartung-Knapp-Sidik-Jonkman adjustment.

Seven studies involving 528 participants were included in the meta-analysis of anxiety [11,12,16,17,20,41,44]. The pooled analysis demonstrated a statistically significant reduction in anxiety, favoring the IVR intervention over control conditions (SMD −0.58, 95% CI −0.95 to −0.20; *P*=.01; Figure 3). Moderate heterogeneity was observed (τ^2^=0.10, *I*^2^=64.2%). The 95% prediction interval was −1.36 to 0.21, indicating that in future similar studies, the true effect may vary from a moderate beneficial effect to a negligible or null effect. One study exhibited a particularly wide CI that crossed the 0 mark, indicating considerable uncertainty in the results [12]. The CIs for the other 2 studies crossed 0, rendering them statistically nonsignificant [11,16]. However, in the study by Kamada et al [12], significant differences were observed in anxiety characteristics between the 2 groups following intervention (*P*<.05). Meanwhile, 2 other RCTs [11,16] reported significant differences in the magnitude of anxiety change between the 2 groups before and after intervention (*P*<.05).

#### Pain

**Figure 4.**
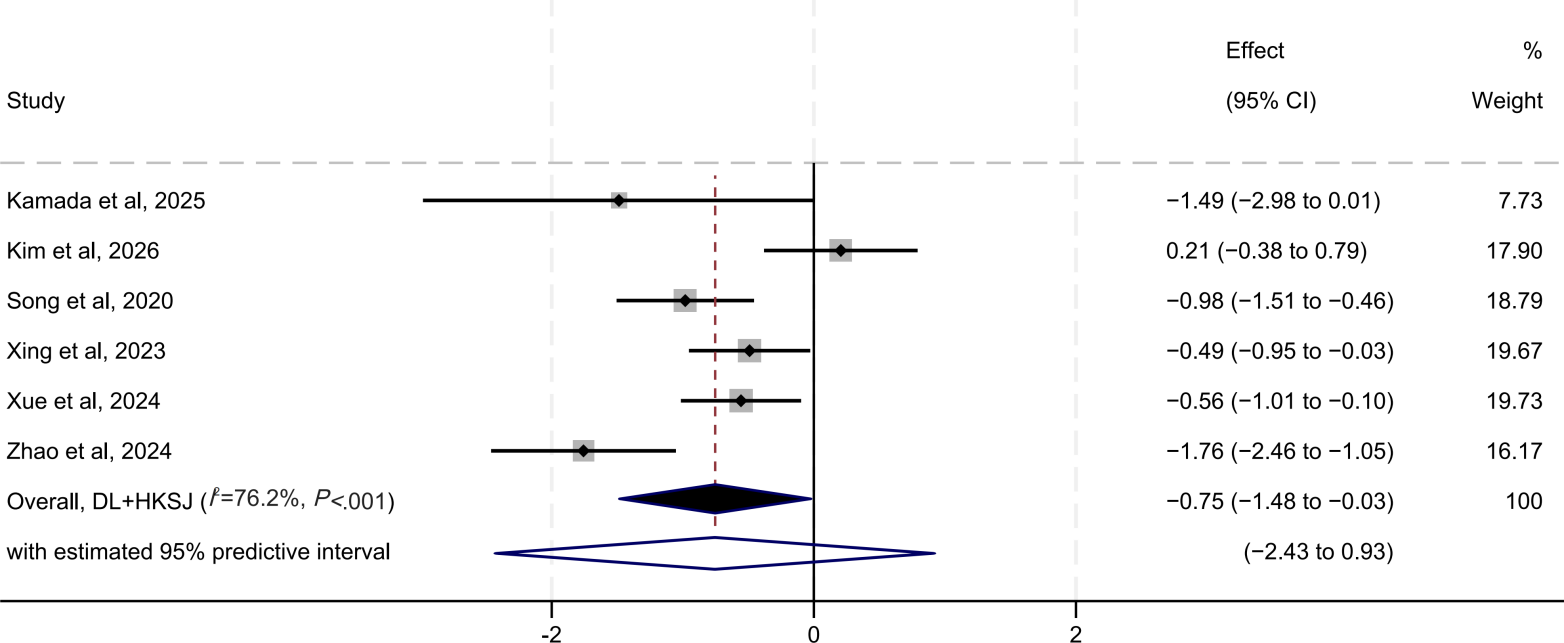
Forest plot of the effect of immersive virtual reality on pain [12,17,39-41,45]. Weights are from a random-effects model. DL+HKSJ: DerSimonian-Laird method with the Hartung-Knapp-Sidik-Jonkman adjustment.

Six studies involving 312 participants were included in the meta-analysis of pain [12,17,39-41,45]. The pooled analysis indicates that immersive VR significantly reduces pain compared with control conditions (SMD −0.75, 95% CI −1.48 to −0.03; *P*=.04). High heterogeneity was observed (τ^2^=0.29, *I*^2^=76.2%). The 95% predicted interval was −2.43 to 0.93, suggesting that the analgesic effect of VR may vary considerably across different clinical settings, from a marked benefit to a negligible or absent effect (Figure 4).

### Effectiveness of Interventions on Secondary Outcome Measures

#### Knowledge

**Figure 5.**
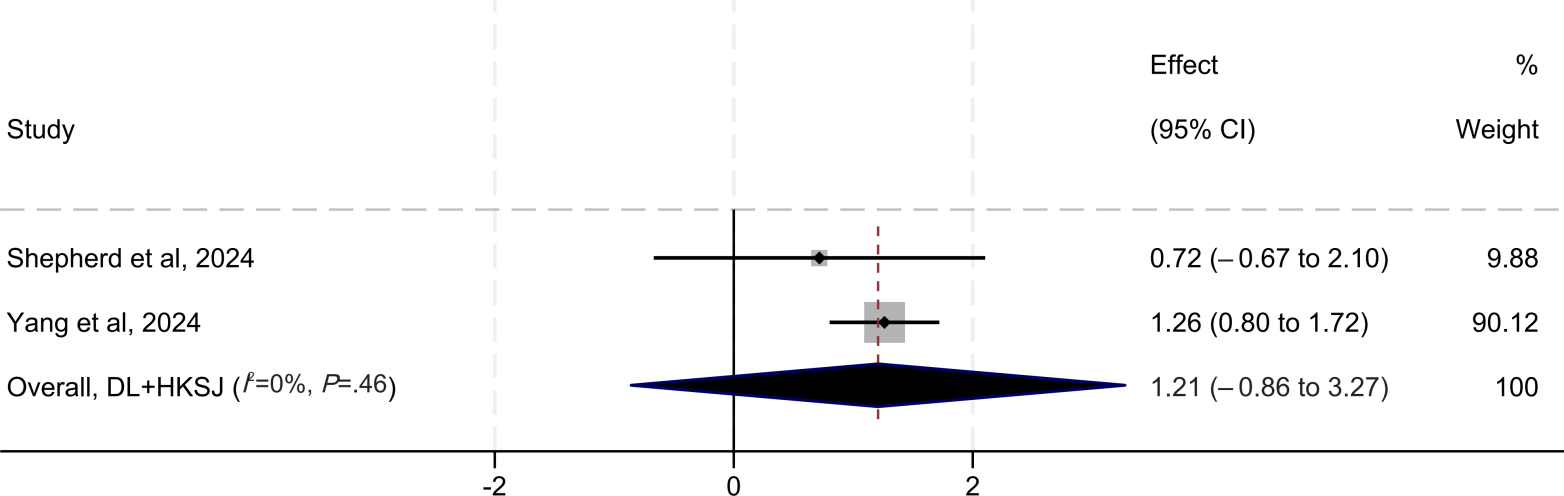
Forest plot of the effect of immersive virtual reality on knowledge [16,42]. Weights are from a random-effects model. DL+HKSJ: DerSimonian-Laird method with the Hartung-Knapp-Sidik-Jonkman adjustment.

Two studies evaluated the impact of IVR on medical knowledge [16,42]. The meta-analysis did not show a statistically significant pooled effect (SMD 1.21, 95% CI −0.86 to 3.27; *P*=.08), although the point estimate favored the intervention (Figure 5). Individually, Yang et al [16] reported that the mean knowledge scores significantly increased by 5.86 (SD 3.7) points following VR-based education, whereas Shepherd et al [42], with a very small sample size, found no significant between-group difference. The clinical relevance of the observed effect size remains uncertain due to the wide CI and limited number of studies. A 95% prediction interval was not calculated for this outcome because fewer than 3 studies were available.

#### Vital Signs

**Figure 6.**
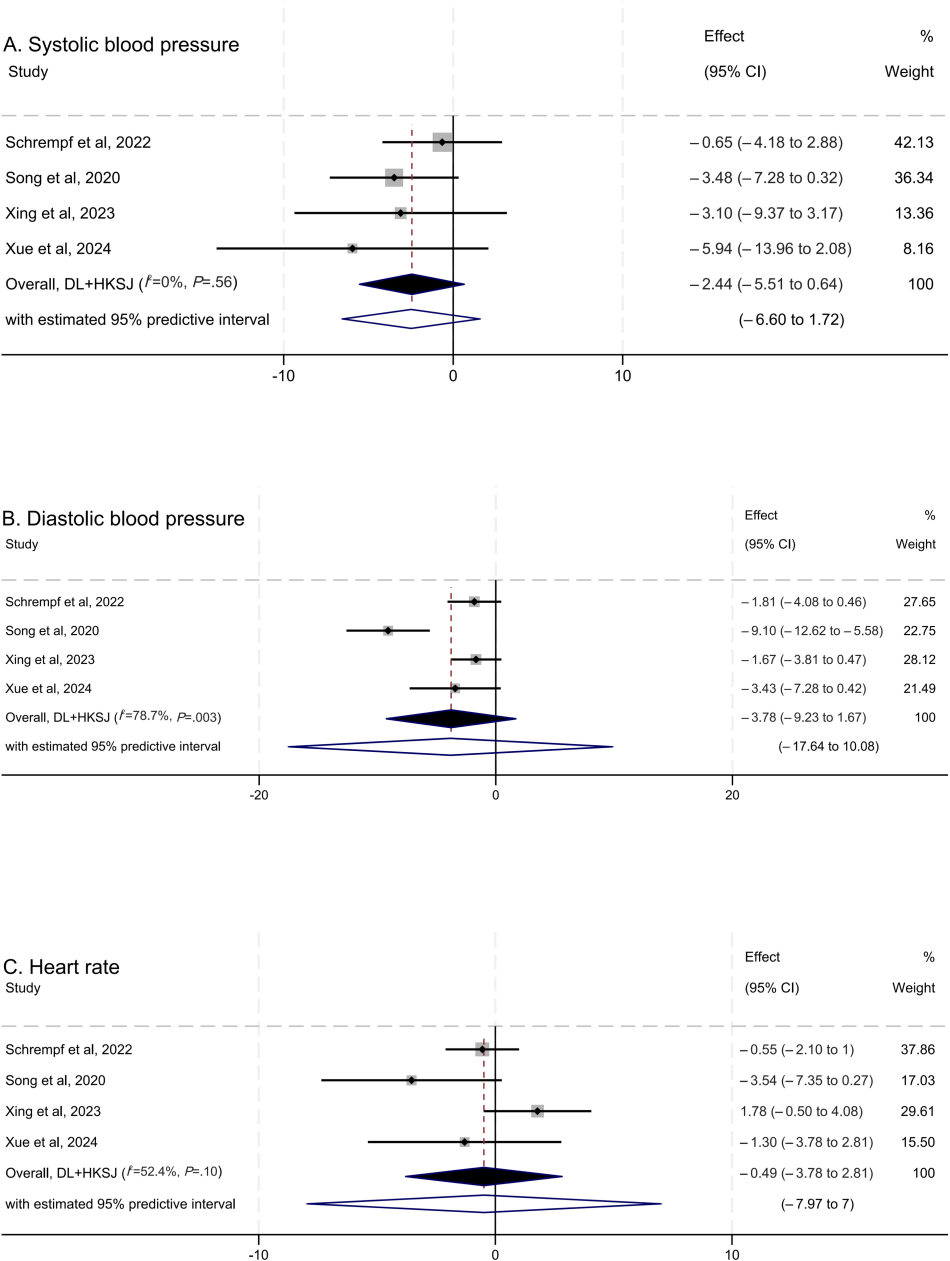
Forest plots of the effect of immersive virtual reality on vital signs: (A) systolic blood pressure, (B) diastolic blood pressure, and (C) heart rate [13,17,40,41]. Weights are from a random-effects model. DL+HKSJ: DerSimonian-Laird method with the Hartung-Knapp-Sidik-Jonkman adjustment.

Four studies involving 250 participants investigated the immediate effects of perioperative IVR on patients’ vital signs [13,17,40,41]. For systolic blood pressure, the pooled effect was not statistically significant (MD −2.44 mm Hg, 95% CI −5.51 to 0.64; *P*=.09; Figure 6A). Heterogeneity was low (τ^2^=0, *I*^2^=0%), and the 95% prediction interval ranged from −6.27 to 2.05, indicating that future studies are likely to observe effects within this window. For diastolic blood pressure, the pooled effect was similarly nonsignificant (MD −3.78 mm Hg, 95% CI −9.23 to 1.67; *P*=.11; Figure 6B). High heterogeneity was observed (τ^2^=7.44, *I*^2^=78.7%), and the 95% prediction interval was extremely wide (−17.63 to 10.08), reflecting considerable uncertainty in the estimated effect across different settings. For heart rate, the overall effect was also not significant (MD −0.49 beats per m, 95% CI −3.78 to 2.81; *P*=.67; Figure 6C), with moderate heterogeneity (τ^2^=1.95, *I*^2^=52.4%). However, Schrempf et al [13] observed a significant difference in heart rate levels within the patient group before and after VR intervention (*P*=.012), with heart rates gradually stabilizing. The remaining 3 studies reported no significant changes in heart rate before and after intervention [17,40,41]. Among the included studies, vital signs served 2 distinct purposes. Three studies used them as objective surrogate measures to assess the emotional improvement effects of IVR interventions [40,41,43]; another study primarily used them as safety indicators to monitor the IVR intervention process [17].

#### Length of Hospital Stay

**Figure 7.**
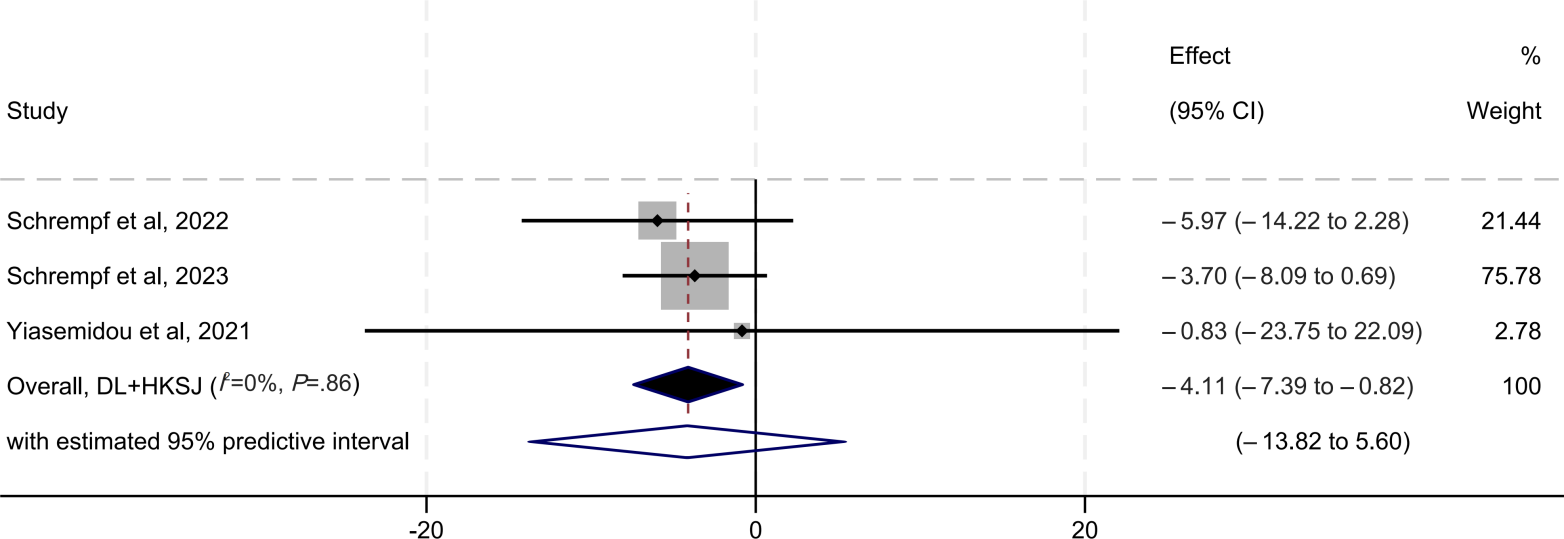
Forest plot of the effect of immersive virtual reality on length of stay [13,43,46]. Weights are from a random-effects model. DL+HKSJ: DerSimonian-Laird method with the Hartung-Knapp-Sidik-Jonkman adjustment.

The pooled analysis of 3 studies [13,43,46] indicated a statistically significant reduction in length of stay associated with IVR (MD −4.11 days, 95% CI −7.39 to −0.82; *P*=.03). Statistical heterogeneity was low (τ^2^=0, *I*^2^=0%). However, the 95% prediction interval was exceptionally wide (−13.82 to 5.60 days), indicating substantial uncertainty about the effect in future clinical settings, which could range from a large reduction to a possible increase in hospitalization (Figure 7).

#### Quality of Life

**Figure 8.**
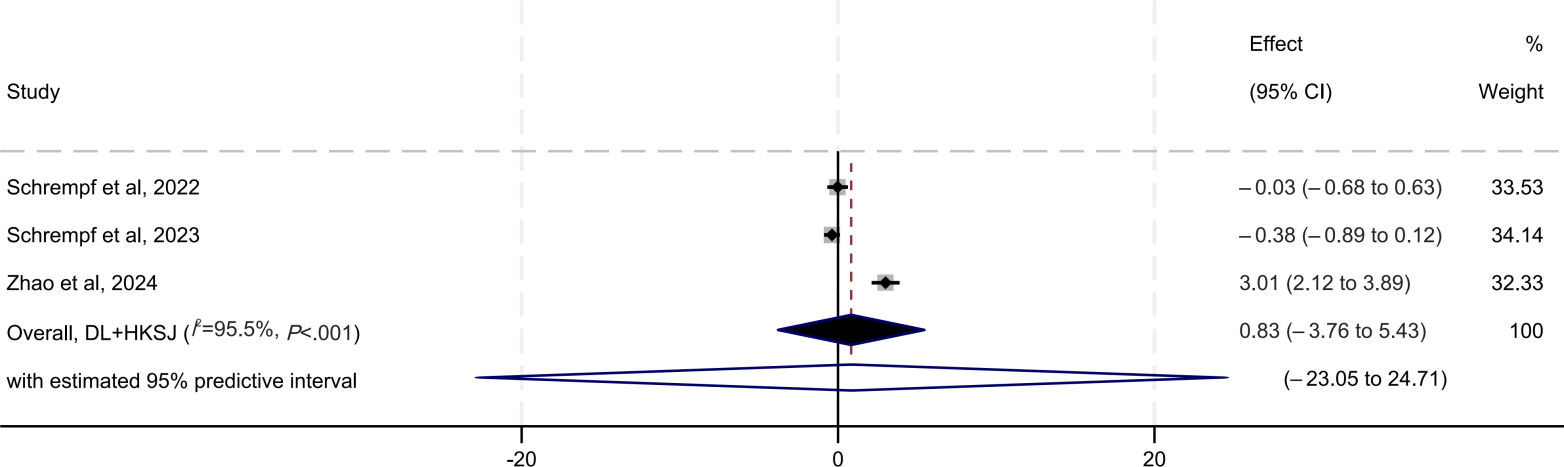
Forest plot of the effect of immersive virtual reality on quality of life [13,39,43]. Weights are from a random-effects model. DL+HKSJ: DerSimonian-Laird method with the Hartung-Knapp-Sidik-Jonkman adjustment.

Three studies comprising 142 participants reported overall quality of life scores using different instruments [13,39,43]. One additional study [11] was excluded from the pooled analysis because the effect size could not be calculated owing to a reported SD of 0 in the control group. Due to high heterogeneity (τ^2^=2.39, *I*^2^=95.5%), the 3 remaining studies were deemed unsuitable for meaningful meta-analysis. The pooled estimate was SMD 0.83 (95% CI −3.76 to 5.43; *P*=.53), indicating no statistically significant difference between the IVR and control groups (Figure 8). In one study [39], patients with esophageal cancer receiving IVR intervention demonstrated superior quality of life compared to the control group (*P*<.001), and over successive chemotherapy cycles, quality of life improved in the intervention group while steadily declining in the control group. The remaining studies all reported no significant difference in quality of life between the intervention and control groups (*P*>.05) [11,13,43].

#### Safety

Four studies examined safety parameters of the intervention [17,42,43,45]. Two studies collected data on patient nausea, vomiting, and dizziness, yet no significant differences were observed [17,45]. The remaining 2 studies reported no adverse reactions to the intervention [42,43].

### Subgroup Analysis and Meta-Regression

**Figure 9.**
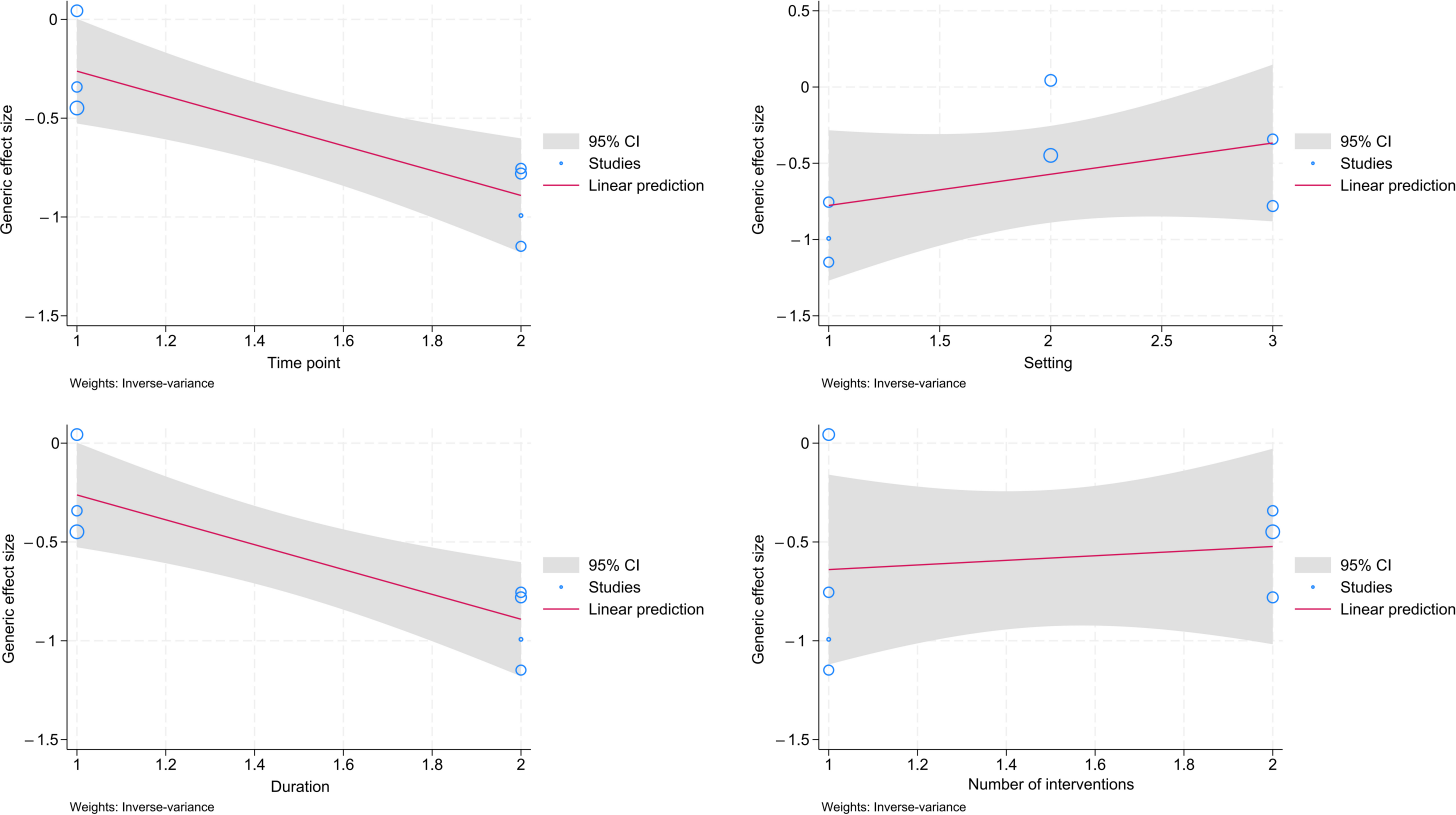
Meta-regression bubble plots for anxiety.

Given the considerable heterogeneity in the effects of IVR interventions on anxiety and pain among patients with gastrointestinal cancer, a series of subgroup analyses and meta-regressions were conducted based on intervention design (Table 3). Regarding anxiety, the time point of the intervention significantly moderated the effect size (between-subgroup *P*=.001; meta-regression *P*=.03). Studies delivering the intervention during treatment yielded a large and statistically significant reduction in anxiety (SMD −0.95, 95% CI −1.32 to −0.46; *P*=.65 within subgroup; *I*^2^=0%), whereas those conducted before treatment showed a smaller, nonsignificant effect (SMD −0.26, 95% CI −0.91 to 0.39; *P*=.20; *I*^2^=38%). A similar situation emerged for intervention duration. Longer interventions lasting ≥20 minutes were associated with a greater anxiolytic effect (SMD −0.95, 95% CI −1.53 to −0.37; *I*^2^=0%) compared with shorter interventions (SMD −0.38, 95% CI −0.92 to 0.16; *I*^2^=58.5%; between-subgroup *P*=.008; meta-regression *P*=.02). Furthermore, the clinical setting also influenced anxiety outcomes (between-subgroup *P*=.02), with the most pronounced benefit observed in operating room settings (SMD −0.95, 95% CI −1.53 to −0.37; *I*^2^=0%). In contrast, the number of intervention sessions did not significantly moderate the effect on anxiety (between-subgroup *P*=.64; meta-regression *P*=.75). However, with regard to pain and diastolic blood pressure, none of the interventions included in the analysis showed a significant difference in outcomes (*P*>.05). The bubble plots are presented in Figures9  10, and the forest plots are provided in Multimedia Appendix 2.

**Table 3.** Subgroup analysis forest plot of the impact of immersive virtual reality interventions on anxiety and pain in patients with gastrointestinal cancer.

Groups	n	*I*^2^ (%)	SMD^a^	95% CI	Within subgroups, *P* value	Between subgroups, *P* value	Meta-regression
Anxiety							
Time point						.001	0.025
Pretreatment	3	38	–0.26	–0.91 to 0.39	.199		
Intratreatment	4	0	–0.95	–1.32 to –0.46	.648		
Setting						.023	0.352
Operating room	3	0	–0.95	–1.53 to –0.37	.523		
Ward	2	67.7	–0.22	–3.34 to 2.91	.079		
Nonmedical setting	2	43	–0.57	–3.35 to 2.21	.185		
Duration						.008	0.025
<20min	4	58.5	–0.38	–0.92 to 0.16	.065		
≥20min	3	0	–0.95	–1.53 to –0.37	.523		
Number						.639	0.753
Once	4	79.5	–0.66	–1.55 to 0.23	.002		
≥Twice	3	1	–0.52	–1.05 to 0.01	.364		
Pain							
Time point						.005	0.248
Pretreatment	1	0	–0.98	–1.51 to –0.46	<.001		
Intratreatment	4	71.8	–0.95	–1.98 to 0.08	.014		
Posttreatment	1	0	0.21	–0.38 to 0.79	<.001		
Setting						.651	0.874
Operating room	3	0	–0.57	–1.18 to 0.05	.458		
Ward	3	89.4	–0.83	–3.27 to 1.61	<.001		
Duration						.412	0.401
<20min	2	88.6	–0.4	–7.93 to 7.14	.003		
≥20min	4	71.8	–0.95	–1.98 to 0.08	.014		
Number						.651	0.874
Once	3	0	–0.57	–1.18 to 0.05	.458		
≥Twice	3	89.4	–0.83	–3.27 to 1.61	<.001		

a SMD: standardized mean difference.

**Figure 10.**
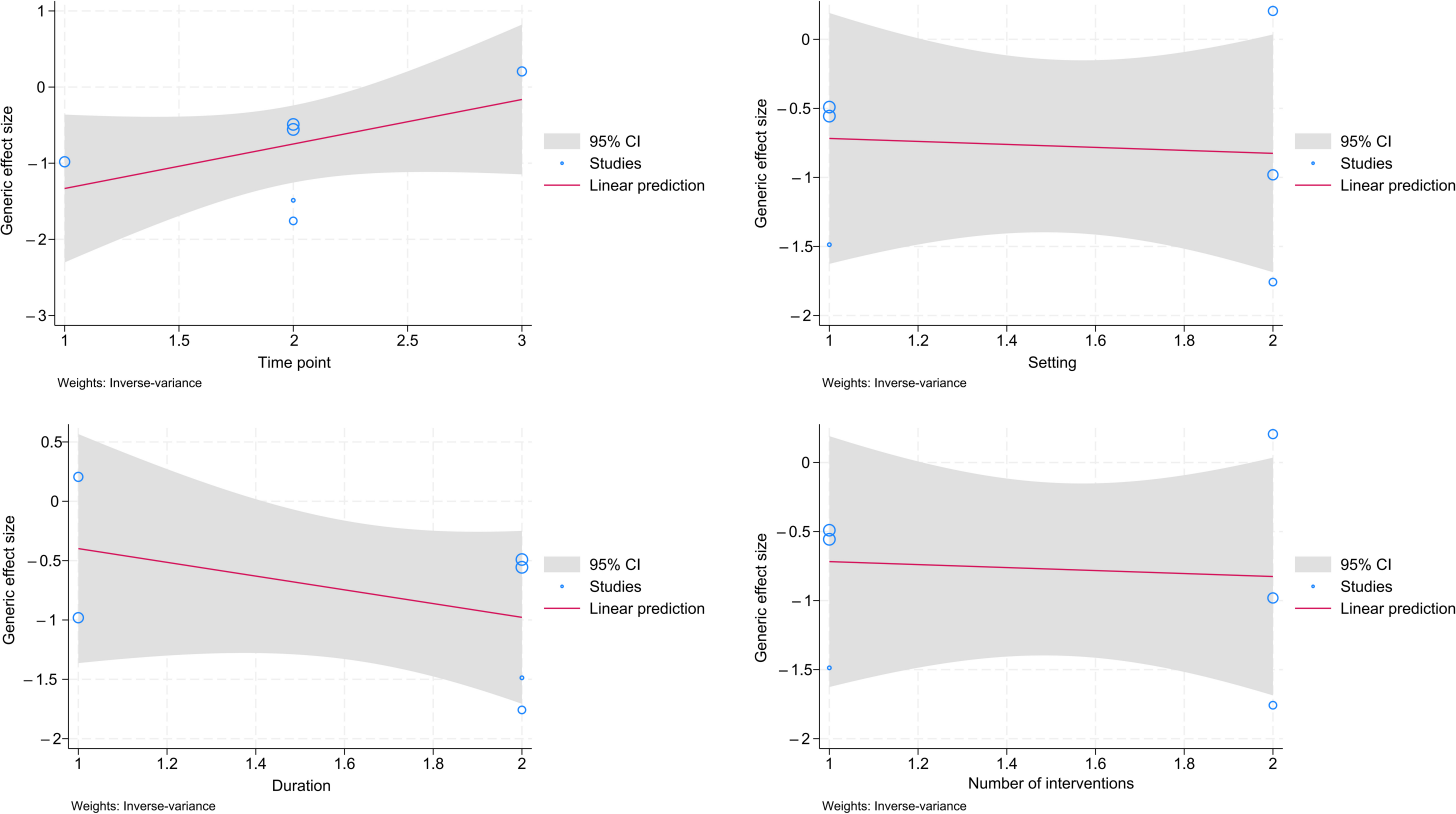
Meta-regression bubble plots for pain.

### Heterogeneity

Heterogeneity among the included studies may have influenced the overall specificity and sensitivity of IVR-based interventions within the internal validation dataset. Using the leave-one-out method, we found the studies to exhibit relatively good stability. We explored sources of heterogeneity through subgroup analyses and meta-regression, ultimately identifying intervention timing and duration as potential factors influencing IVR’s efficacy in alleviating patient anxiety. Sensitivity analyses, conducted by sequentially omitting individual studies, revealed that the pooled effects across most iterations remained statistically significant, demonstrating relatively high degrees of robustness. For further details, please refer to Multimedia Appendix 2.

## Discussion

### Principal Findings

This systematic review and meta-analysis synthesized evidence from 14 RCTs involving 837 patients to evaluate the effectiveness of IVR interventions for symptom management in patients with gastrointestinal cancer. The pooled analyses demonstrated that IVR significantly reduced anxiety and pain, which were 2 core symptoms targeted by symptom management interventions. In addition, IVR was associated with a significant reduction in length of hospital stay. No statistically significant effects were observed for quality of life, symptom management knowledge, or vital signs. However, the interpretation of these findings requires careful consideration of the underlying heterogeneity, risk of bias, and certainty of evidence. Specifically, while the 95% CIs indicated that the average effect of IVR on anxiety, pain, and length of stay was statistically significant, the corresponding 95% prediction intervals were wide and included the null value for all 3 outcomes. This discrepancy underscores that the true effect of IVR in a future clinical setting may vary substantially, depending on contextual factors such as intervention timing, session duration, and patient characteristics. Furthermore, the GRADE certainty of evidence was rated as low for anxiety and pain and very low for quality of life, with downgrades primarily attributable to serious risk of bias, inconsistency, and imprecision. Consequently, these findings should be interpreted as promising but preliminary.

### Comparison With Previous Literature

The significant reductions in anxiety and pain observed in this review are broadly consistent with prior systematic reviews examining VR-based interventions in cancer populations [18,19]. For instance, Wu et al [18] reported moderate effects of VR on both anxiety and pain across various cancer types. The present review extends these findings by demonstrating that IVR remains effective specifically in the gastrointestinal cancer population, a group that faces unique symptom challenges including visceral pain and heightened situational anxiety during procedures such as transarterial chemoembolization [47,48].

The subgroup analyses indicates that the time point and duration of intervention are critical factors influencing anxiety levels. Interventions implemented during treatment, such as local anesthesia surgery [12] or chemotherapy [17,41], were associated with a large and statistically significant reduction in anxiety. This situation aligns with the attentional mechanisms proposed by Usman et al [49

] and may be particularly relevant for patients undergoing transarterial chemoembolization. During transarterial chemoembolization, patients remain conscious throughout a protracted sequence of invasive maneuvers, such as vascular puncture, which collectively drive anxiety to peak levels during the procedure itself [47,48]. In this context, highly immersive VR engages multiple sensory channels simultaneously, diverting attentional resources away from noxious stimuli and psychologically transporting the patient from the stressful treatment environment into a controlled, calming virtual space [19]. By directly interrupting the ongoing stressors, IVR exerts a more immediate and potent emotional regulatory effect than interventions delivered prior to treatment [50]. Pretreatment applications, such as simulated procedural walkthroughs [44] or knowledge-based education [16], primarily address the cognitive dimension of anticipatory anxiety. They struggle to fully replicate the complex multisensory stimuli of the actual treatment setting, such as equipment noise, physical discomfort, and antiseptic odors, and the resulting discrepancy between expectation and reality may paradoxically heighten anxiety once the procedure commences [51]. With respect to intervention duration, sessions lasting 20 minutes or longer produced significantly greater anxiety reduction than shorter sessions. This finding is consistent with theoretical models positing a neural adaptation window of approximately 20 minutes for transitioning from an aroused sympathetic state to a parasympathetically mediated relaxed state [6,49,52]. A longer duration permits patients to fully acclimatize to the virtual environment, establish a sense of presence, and engage deeply with the intervention content, which is particularly crucial for adult patients [53]. Nevertheless, the benefits of prolonged exposure appear subject to diminishing returns. Evidence suggests that beyond 30 minutes per session, additional anxiolytic gains plateau and may even reverse due to visual fatigue or physical discomfort associated with prolonged headset wear [52,54]. Although this systematic review could not determine the optimal session frequency, related evidence suggests that training 3 to 5 times weekly may enhance patients’ ability to apply relaxation techniques learned in virtual environments, thereby promoting skill consolidation and sustained symptom self-management [52,54]. This hypothesis warrants prospective validation in future trials.

With respect to pain, neither subgroup analysis nor meta-regression identified statistically significant moderators of the IVR effect. Nevertheless, a sensitivity analysis pointed to a likely source of the observed heterogeneity. Exclusion of the study by Zhao et al [39] reduced τ^2^ from 0.16 to 0.01 and *I*^2^ from 76.2% to 9%. This study differed notably from the others in both intervention duration and pain phenotype. Zhao et al [39] evaluated a 2-month multimodal IVR program designed to manage chronic pain and associated symptoms during chemotherapy for esophageal cancer. In contrast, the remaining studies examined single-session, short-term IVR protocols targeting acute procedural pain during surgery or localized interventions [12,17,41]. This situation suggests that IVR may operate through distinct mechanisms in acute versus chronic pain contexts. Acute procedural pain relief likely depends on immediate attentional distraction, whereas chronic cancer pain, which frequently involves central sensitization and psychological comorbidity [55], may require extended multimodal interventions that foster sustained emotional regulation and enhanced self-efficacy. Whether these differential effects account for the apparent inconsistency with prior reviews reporting VR benefits predominantly in acute pain settings [56] warrants further investigation.

With respect to symptom management knowledge, the pooled analysis did not reveal a statistically significant benefit of IVR, which contrasts with studies of mobile health and telemedicine interventions that have demonstrated significant improvements in disease-related knowledge among patients with cancer [57,58]. This discrepancy may reflect a difference in intervention design focus. Unlike digital health platforms specifically engineered for structured knowledge transfer, the IVR interventions included in this review primarily emphasized immersive relaxation and attention distraction [51]. The experiential, rather than instructional, orientation of these applications may limit their ability to convey factual information about symptom identification, self-monitoring, and coping strategies [59]. Nevertheless, the wide CI and moderate GRADE certainty suggest that a potential benefit cannot be excluded. Whether integrating educational modules within immersive symptom management environments enhances knowledge acquisition merits further study.

This meta-analysis indicates that perioperative IVR interventions exert no significant immediate effect on vital signs in patients with gastrointestinal cancer, consistent with findings from prior studies [60]. Notably, stable vital signs are frequently regarded as a physiological reflection of the patient’s psychological equilibrium. Multiple studies indicate that IVR aids in maintaining intraoperative vital sign stability, reflecting both its role in emotional regulation [6,61] and providing evidence for its safety as an adjunctive intraoperative intervention [17]. Consequently, despite failing to achieve statistical significance, IVR’s performance in sustaining vital sign stability supports its feasibility and safety for perioperative application.

The pooled reduction in length of hospital stay observed in this review is consistent with the hypothesis that effective symptom management may accelerate postoperative recovery and facilitate earlier discharge [46,55]. Comparable reductions in hospitalization have been reported in reviews of VR-based rehabilitation in surgical populations [43]. However, this finding must be interpreted with considerable caution. The 95% prediction interval was exceptionally wide, reflecting substantial uncertainty about the expected effect in different health care settings or patient populations. Moreover, the GRADE certainty of evidence was downgraded to moderate due to serious imprecision arising from the limited cumulative sample size. While the observed effect is clinically promising, it should be viewed as hypothesis generating rather than definitive.

This meta-analysis indicates that the efficacy of IVR interventions in improving patient quality of life remains inconclusive. This uncertainty likely stems primarily from methodological limitations in the design and reporting of end point measures across existing studies. Patient quality of life constitutes a multidimensional long-term end point, with significant improvements typically requiring cumulative temporal effects [62]. However, the studies included in this analysis predominantly used single-session or short-term intervention protocols and systematically lacked comprehensive long-term follow-up data. Consequently, the existing evidence base may be insufficient to capture the potential long-term impact of IVR on quality of life. These findings echo those of Hao et al [15], whose umbrella review noted that evidence for VR effects on quality of life in cancer populations is sparse and methodologically weak.

Regarding safety, our research indicates that IVR was well tolerated by patients, contrasting with concerns raised by Tian et al [63]. This discrepancy may stem from our included studies primarily relying on patient self-reported adverse reactions. The majority of enrolled patients were aged over 60 years, and susceptibility to motion sickness typically diminishes with advancing age [18]. One study analyzed patients’ motion sickness responses by measuring blood pressure and heart rate [17], echoing findings by Chattha et al [64] and implying a potential correlation between physiological variables and motion sickness.

### Limitations

Several limitations should be considered in this meta-analysis. First, the number of included studies was relatively small for certain outcomes (eg, knowledge), which may limit the statistical power and generalizability of these findings. Second, the overall risk of bias was rated as high or of some concern in more than half of the included studies, particularly in the domains of randomization and blinding, which may affect the validity of the pooled estimates. Third, certain clinical and methodological heterogeneity across studies, along with the low to moderate certainty of evidence for most outcomes as assessed by GRADE, suggests that the results should be interpreted with caution and that firmer conclusions await more consistent and precise data. Finally, as only articles published in English and Chinese were included, language bias cannot be entirely ruled out. These limitations highlight the need for larger, more rigorously designed RCTs with standardized intervention protocols and outcome measures to further validate and extend our findings.

### Conclusion

This systematic review and meta-analysis provides evidence that IVR is an effective nonpharmacological adjunct for symptom management in patients with gastrointestinal cancer, significantly reducing anxiety and pain as 2 core symptoms in this population and potentially shortening hospital stay. Subgroup analyses indicate that these benefits are maximized when IVR is administered during active treatment and for session durations of at least 20 minutes, offering actionable parameters for clinical implementation. However, the GRADE certainty of evidence was rated as low for anxiety and pain, and the wide 95% prediction intervals underscore substantial uncertainty about effect magnitudes across different clinical settings. Consequently, these findings should be interpreted as promising but preliminary. Future research should prioritize well-powered, multicenter trials with standardized protocols and longer-term follow-up to confirm the durability of symptom management benefits and validate optimal intervention parameters.

## Supplementary material

10.2196/86808 Multimedia Appendix 1 Search strategy.

10.2196/86808 Multimedia Appendix 2 Details of forest plots for subgroup analyses and sensitivity analyses.

10.2196/86808 Checklist 1 PRISMA (Preferred Reporting Items for Systematic Reviews and Meta-Analyses) 2020 checklist.

10.2196/86808 Checklist 2 PRISMA (Preferred Reporting Items for Systematic Reviews and Meta-Analyses) 2020 for Abstracts checklist.

10.2196/86808 Checklist 3 PRISMA-S (Preferred Reporting Items for Systematic Reviews and Meta-Analyses literature search extension) checklist.

## Abbreviations

CBM: China Biology Medicine disc
CENTRAL: Cochrane Central Register of Controlled Trials
CNKI: China National Knowledge Infrastructure
GRADE: Grading of Recommendations, Assessment, Development, and Evaluation
IVR: immersive virtual reality
PI: prediction interval
PRISMA: Preferred Reporting Items for Systematic Reviews and Meta-Analyses
PRISMA-S: Preferred Reporting Items for Systematic Reviews and Meta-Analyses literature search extension
RCT: randomized controlled trial
SMD: standardized mean difference
VR: virtual reality
