# Supplementary material for: Effects of Immersive Virtual Reality Interventions on Symptom Management in Patients With Gastrointestinal Cancer: Systematic Review and Meta-Analysis of Randomized Controlled Trials
Source: J Med Internet Res. 2026 Jul 2;28:e86808. doi: 10.2196/86808 (PMC13327534; doi:10.2196/86808)
Supplement: Multimedia Appendix 1 [file jmir-v28-e86808-s001.docx]

**Multimedia Appendix 1.** Search strategy

| Database | # | Search terms | Results |
| --- | --- | --- | --- |
| Pubmed  (NLM) | #1 | "gastrointestinal neoplasms"[MeSH Terms] OR "digestive system neoplasms"[MeSH Terms] | 807,790 |
|  | #2 | (GI[Title/Abstract] OR gastrointestinal[Title/Abstract] OR digestive[Title/Abstract] OR esopha*[Title/Abstract] OR oesophag*[Title/Abstract] OR gastric[Title/Abstract] OR stomach[Title/Abstract] OR pancrea*[Title/Abstract] OR Liver[Title/Abstract] OR hepat*[Title/Abstract] OR colorectal[Title/Abstract] OR colon*[Title/Abstract] OR rect*[Title/Abstract] OR bowel[Title/Abstract] OR sigmoid[Title/Abstract] OR intestin*[Title/Abstract] OR duodenal[Title/Abstract] OR jejunal[Title/Abstract] OR ileal[Title/Abstract] OR biliary[Title/Abstract] OR gallbladder[Title/Abstract] OR cholangio*[Title/Abstract]) AND (cancer[Title/Abstract] OR neoplas*[Title/Abstract] OR tumor[Title/Abstract] OR tumour*[Title/Abstract] OR carcinoma[Title/Abstract] OR malignanc*[Title/Abstract] OR adenocarcinoma[Title/Abstract] OR carcinoid[Title/Abstract] OR lymphoma[Title/Abstract]) | 1,184,375 |
|  | #3 | #1 OR #2 | 1,359,964 |
|  | #4 | "virtual reality"[MeSH Terms] OR "virtual reality exposure therapy"[MeSH Terms] OR "smart glasses" [MeSH Terms] | 29,689 |
|  | #5 | "virtual realit*"[Title/Abstract] OR VR[Title/Abstract] OR "virtual environment"[Title/Abstract] OR "virtual treatment"[Title/Abstract] OR immersive[Title/Abstract] OR "virtual care"[Title/Abstract] OR simulation[Title/Abstract] OR "virtual medicine"[Title/Abstract] OR "smart glass*"[Title/Abstract] OR "head up display"[Title/Abstract] OR "head mounted display"[Title/Abstract] OR "Google Glass"[Title/Abstract] OR Kinect[Title/Abstract] OR Wii[Title/Abstract] OR hmd[Title/Abstract] OR Oculus[Title/Abstract] | 390,880 |
|  | #6 | #4 OR #5 | 392,571 |
|  | #7 | randomized controlled trial[MeSH Terms] | 199,883 |
|  | #8 | randomized controlled trial[Title/Abstract] OR controlled clinical trial[Title/Abstract] OR randomized[Title/Abstract] OR randomly[Title/Abstract] | 1,209,709 |
|  | #9 | #7 OR #8 | 1,302,742 |
|  | #10 | #3 AND #6 AND #9 | 267 |
| Scopus  (Elsevier) | #1 | ( TITLE-ABS-KEY ( ( GI OR gastrointestinal OR digestive OR esopha* OR oesophag* OR gastric OR stomach OR pancrea* OR Liver OR hepat* OR colorectal OR colon* OR rect* OR bowel OR sigmoid OR intestin* OR duodenal OR jejunal OR ileal OR biliary OR gallbladder OR cholangio* ) AND ( cancer OR neoplas* OR tumor OR tumour* OR carcinoma OR malignanc* OR adenocarcinoma OR carcinoid OR lymphoma ) ) AND TITLE-ABS-KEY ( "virtual realit*" OR VR OR "virtual environment" OR "virtual treatment" OR immersive OR "virtual care" OR simulation OR "virtual medicine" OR "smart glass*" OR "head up display" OR "head mounted display" OR "Google Glass" OR Kinect OR Wii OR hmd OR Oculus ) AND TITLE-ABS-KEY ( randomized controlled trial OR controlled clinical trial OR randomized OR randomly ) ) | 455 |
| Web of Science  (Clarivate Analytics) | #1 | TS=( ( GI OR gastrointestinal OR digestive OR esopha* OR oesophag* OR gastric OR stomach OR pancrea* OR Liver OR hepat* OR colorectal OR colon* OR rect* OR bowel OR sigmoid OR intestin* OR duodenal OR jejunal OR ileal OR biliary OR gallbladder OR cholangio* ) AND ( cancer OR neoplas* OR tumor OR tumour* OR carcinoma OR malignanc* OR adenocarcinoma OR carcinoid OR lymphoma ) ) and Preprint Citation Index (Exclude – Database) | 1,514,577 |
|  | #2 | TS=("virtual realit*" OR VR OR "virtual environment" OR "virtual treatment" OR immersive OR "virtual care" OR simulation OR "virtual medicine" OR "smart glass*" OR "head up display" OR "head mounted display" OR "Google Glass" OR Kinect OR Wii OR hmd OR Oculus) and Preprint Citation Index (Exclude – Database) | 4,152,792 |
|  | #3 | TS=(randomized controlled trial OR controlled clinical trial OR randomized OR randomly) and Preprint Citation Index (Exclude – Database) | 1,867,900 |
|  | #4 | #1 AND #2 AND #3 and Preprint Citation Index (Exclude – Database) | 591 |
| Cochrane Central Register of Controlled Trials  (Cochrane Library) | #1 | MeSH descriptor: [Gastrointestinal Neoplasms] explode all trees | 21,268 |
|  | #2 | MeSH descriptor: [Digestive System Neoplasms] explode all trees | 28,854 |
|  | #3 | ((GI OR gastrointestinal OR digestive OR esopha* OR oesophag* OR gastric OR stomach OR pancrea* OR Liver OR hepat* OR colorectal OR colon* OR rect* OR bowel OR sigmoid OR intestin* OR duodenal OR jejunal OR ileal OR biliary OR gallbladder OR cholangio*) AND (cancer OR neoplas* OR tumor OR tumour* OR carcinoma OR malignanc* OR adenocarcinoma OR carcinoid OR lymphoma)):ti,ab,kw | 99,242 |
|  | #4 | #1 OR #2 OR #3 | 99,743 |
|  | #5 | MeSH descriptor: [Virtual Reality] explode all trees | 1,829 |
|  | #6 | MeSH descriptor: [Virtual Reality Exposure Therapy] explode all trees | 466 |
|  | #7 | MeSH descriptor: [Smart Glasses] explode all trees | 31 |
|  | #8 | ((Virtual NEXT Realit*) OR VR OR "virtual environment" OR "virtual treatment" OR immersive OR "virtual care" OR simulation OR "virtual medicine" OR (smart NEXT glass*) OR "head up display" OR "head mounted display" OR "google glass" OR kinect OR wii OR hmd OR oculus):ti,ab,kw | 53,077 |
|  | #9 | #8 OR #9 OR #10 OR #11 | 53,095 |
|  | #10 | MeSH descriptor: [Randomized Controlled Trial] explode all trees | 34 |
|  | #11 | randomized controlled trial OR controlled clinical trial OR randomized OR randomly | 1,566,468 |
|  | #12 | #10 OR #11 | 1,566,470 |
|  | #13 | #4 AND #9 AND #12 | 775 |
| CINAHL  (EBSCOhost) | #1 | MH ("gastrointestinal neoplasms" OR "digestive system neoplasms") OR XB ((GI OR gastrointestinal OR digestive OR esopha* OR oesophag* OR gastric OR stomach OR pancrea* OR Liver OR hepat* OR colorectal OR colon* OR rect* OR bowel OR sigmoid OR intestin* OR duodenal OR jejunal OR ileal OR biliary OR gallbladder OR cholangio*) AND (cancer OR neoplas* OR tumor OR tumour* OR carcinoma OR malignanc* OR adenocarcinoma OR carcinoid OR lymphoma)) | 171,336 |
|  | #2 | MH ("virtual reality" OR "virtual reality exposure therapy" OR "smart glasses") OR XB ("virtual realit*" OR VR OR "virtual environment" OR "virtual treatment" OR immersive OR "virtual care" OR simulation OR "virtual medicine" OR "smart glass*" OR "head up display" OR "head mounted display" OR "Google Glass" OR Kinect OR Wii OR hmd OR Oculus) | 64,393 |
|  | #3 | MH (randomized controlled trial) OR XB (randomized controlled trial OR controlled clinical trial OR randomized OR randomly) | 426,682 |
|  | #4 | #1 AND #2 AND #3 | 76 |
| Embase  (Elsevier) | #1 | 'gastrointestinal tumor'/exp OR 'digestive system tumor'/exp OR ((gi:ti,ab,kw OR gastrointestinal:ti,ab,kw OR digestive:ti,ab,kw OR esopha*:ti,ab,kw OR oesophag*:ti,ab,kw OR gastric:ti,ab,kw OR stomach:ti,ab,kw OR pancrea*:ti,ab,kw OR liver:ti,ab,kw OR hepat*:ti,ab,kw OR colorectal:ti,ab,kw OR colon*:ti,ab,kw OR rect*:ti,ab,kw OR bowel:ti,ab,kw OR sigmoid:ti,ab,kw OR intestin*:ti,ab,kw OR duodenal:ti,ab,kw OR jejunal:ti,ab,kw OR ileal:ti,ab,kw OR biliary:ti,ab,kw OR gallbladder:ti,ab,kw OR cholangio*:ti,ab,kw) AND (cancer:ti,ab,kw OR neoplas*:ti,ab,kw OR tumor:ti,ab,kw OR tumour*:ti,ab,kw OR carcinoma:ti,ab,kw OR malignanc*:ti,ab,kw OR adenocarcinoma:ti,ab,kw OR carcinoid:ti,ab,kw OR lymphoma:ti,ab,kw)) | 2,155,666 |
|  | #2 | 'virtual reality'/exp OR 'virtual reality exposure therapy'/exp OR 'smart glasses'/exp OR 'virtual realit*':ti,ab,kw OR vr:ti,ab,kw OR 'virtual environment':ti,ab,kw OR 'virtual treatment':ti,ab,kw OR immersive:ti,ab,kw OR 'virtual care':ti,ab,kw OR simulation:ti,ab,kw OR 'virtual medicine':ti,ab,kw OR 'smart glass*':ti,ab,kw OR 'head up display':ti,ab,kw OR 'head mounted display':ti,ab,kw OR 'google glass':ti,ab,kw OR kinect:ti,ab,kw OR wii:ti,ab,kw OR hmd:ti,ab,kw OR oculus:ti,ab,kw | 458,183 |
|  | #3 | 'randomized controlled trial'/exp OR 'randomized controlled trial':ti,ab,kw OR 'controlled clinical trial':ti,ab,kw OR randomized:ti,ab,kw OR randomly:ti,ab,kw | 2,215,627 |
|  | #4 | #1 AND #2 AND #3 | 676 |
| PsycINFO  (EBSCOhost) | #1 | MH ("gastrointestinal neoplasms" OR "digestive system neoplasms") OR XB ((GI OR gastrointestinal OR digestive OR esopha* OR oesophag* OR gastric OR stomach OR pancrea* OR Liver OR hepat* OR colorectal OR colon* OR rect* OR bowel OR sigmoid OR intestin* OR duodenal OR jejunal OR ileal OR biliary OR gallbladder OR cholangio*) AND (cancer OR neoplas* OR tumor OR tumour* OR carcinoma OR malignanc* OR adenocarcinoma OR carcinoid OR lymphoma)) | 10,629 |
|  | #2 | MH ("virtual reality" OR "virtual reality exposure therapy" OR "smart glasses") OR XB ("virtual realit*" OR VR OR "virtual environment" OR "virtual treatment" OR immersive OR "virtual care" OR simulation OR "virtual medicine" OR "smart glass*" OR "head up display" OR "head mounted display" OR "Google Glass" OR Kinect OR Wii OR hmd OR Oculus) | 77,775 |
|  | #3 | MH (randomized controlled trial) OR XB (randomized controlled trial OR controlled clinical trial OR randomized OR randomly) | 216,114 |
|  | #4 | #1 AND #2 AND #3 | 11 |
| IEEE Xplore  (IEEE) | #1 | ((GI OR gastrointestinal OR digestive OR esopha* OR oesophag* OR gastric OR stomach OR pancrea* OR Liver OR hepat* OR colorectal OR colon* OR rect* OR bowel OR sigmoid OR intestin* OR duodenal OR jejunal OR ileal OR biliary OR gallbladder OR cholangio*) AND (cancer OR neoplas* OR tumor OR tumour* OR carcinoma OR malignanc* OR adenocarcinoma OR carcinoid OR lymphoma)) AND ("virtual realit*" OR VR OR "virtual environment" OR "virtual treatment" OR immersive OR "virtual care" OR simulation OR "virtual medicine" OR "smart glass*" OR "head up display" OR "head mounted display" OR "Google Glass" OR Kinect OR Wii OR hmd OR Oculus) AND (randomized controlled trial OR controlled clinical trial OR randomized OR randomly) | 25 |
| ProQuest Dissertations and Theses Global  (ProQuest) | #1 | mainsubject("gastrointestinal neoplasms" OR "digestive system neoplasms") OR title((GI OR gastrointestinal OR digestive OR esopha* OR oesophag* OR gastric OR stomach OR pancrea* OR Liver OR hepat* OR colorectal OR colon* OR rect* OR bowel OR sigmoid OR intestin* OR duodenal OR jejunal OR ileal OR biliary OR gallbladder OR cholangio*) AND (cancer OR neoplas* OR tumor OR tumour* OR carcinoma OR malignanc* OR adenocarcinoma OR carcinoid OR lymphoma)) OR abstract((GI OR gastrointestinal OR digestive OR esopha* OR oesophag* OR gastric OR stomach OR pancrea* OR Liver OR hepat* OR colorectal OR colon* OR rect* OR bowel OR sigmoid OR intestin* OR duodenal OR jejunal OR ileal OR biliary OR gallbladder OR cholangio*) AND (cancer OR neoplas* OR tumor OR tumour* OR carcinoma OR malignanc* OR adenocarcinoma OR carcinoid OR lymphoma)) | 37,203 |
|  | #2 | mainsubject("virtual reality" OR "virtual reality exposure therapy" OR "smart glasses") OR title("virtual realit*" OR VR OR "virtual environment" OR "virtual treatment" OR immersive OR "virtual care" OR simulation OR "virtual medicine" OR "smart glass*" OR "head up display" OR "head mounted display" OR "Google Glass" OR Kinect OR Wii OR hmd OR Oculus) OR abstract("virtual realit*" OR VR OR "virtual environment" OR "virtual treatment" OR immersive OR "virtual care" OR simulation OR "virtual medicine" OR "smart glass*" OR "head up display" OR "head mounted display" OR "Google Glass" OR Kinect OR Wii OR hmd OR Oculus) | 328,262 |
|  | #3 | mainsubject(randomized controlled trial) OR title(randomized controlled trial OR controlled clinical trial OR randomized OR randomly) OR abstract(randomized controlled trial OR controlled clinical trial OR randomized OR randomly) | 108,238 |
|  | #4 | #1 AND #2 AND #3 | 11 |
| CNKI | #1 | （主题：（食管 + 胃 + 胰腺 + 肝 + 胆 + 肠）AND（癌 + 肿瘤））AND（主题：虚拟现实 + 头戴式设备 + 沉浸式） | 26 |
| Wangfang Database | #1 | 主题:(癌 OR 肿瘤) and 主题:(虚拟现实 OR 头戴式设备 OR 沉浸式) 过滤器：期刊论文; 学位论文 | 87 |
| VIP | #1 | (((((((题名或关键词=食管 OR 题名或关键词=胃) OR 题名或关键词=胰腺) OR 题名或关键词=肝) OR 题名或关键词=胆) OR 题名或关键词=肠) AND (题名或关键词=癌 OR 题名或关键词=肿瘤)) AND ((题名或关键词=虚拟现实 OR 题名或关键词=头戴式设备) OR 题名或关键词=沉浸式)) | 6 |
| CBM | #1 | (( "食管"[常用字段:智能] OR "胃"[常用字段:智能] OR "胰腺"[常用字段:智能] OR "肝"[常用字段:智能] OR "胆"[常用字段:智能] OR "肠"[常用字段:智能]) AND( "癌"[常用字段:智能] OR "肿瘤"[常用字段:智能])) AND (( "虚拟现实"[常用字段:智能] OR "头戴式设备"[常用字段:智能] OR "沉浸式"[常用字段:智能])) | 59 |

Note: According to the EBSCOhost platform conventions for CINAHL & PsycINFO, the field code “XB” denotes “Title OR Abstract”.
