# Supplementary material for: Effects of Immersive Virtual Reality Interventions on Symptom Management in Patients With Gastrointestinal Cancer: Systematic Review and Meta-Analysis of Randomized Controlled Trials
Source: J Med Internet Res. 2026 Jul 2;28:e86808. doi: 10.2196/86808 (PMC13327534; doi:10.2196/86808)
Supplement: Multimedia Appendix 2 [file jmir-v28-e86808-s002.docx]

1. **Forest plot for subgroup analysis**
   1. **Anxiety**
      1. **Time point**

**
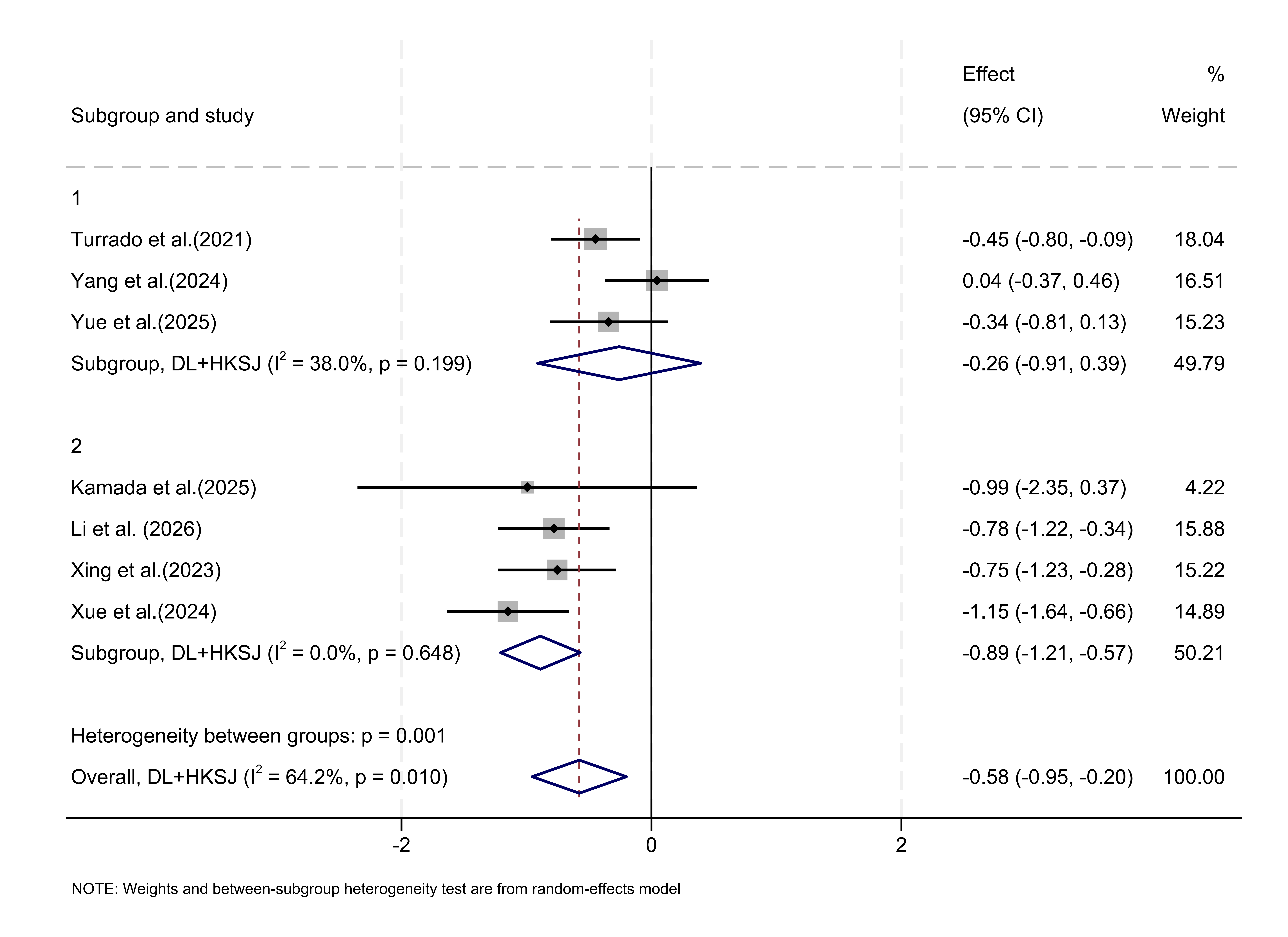
**

- - 1. **Setting**

**
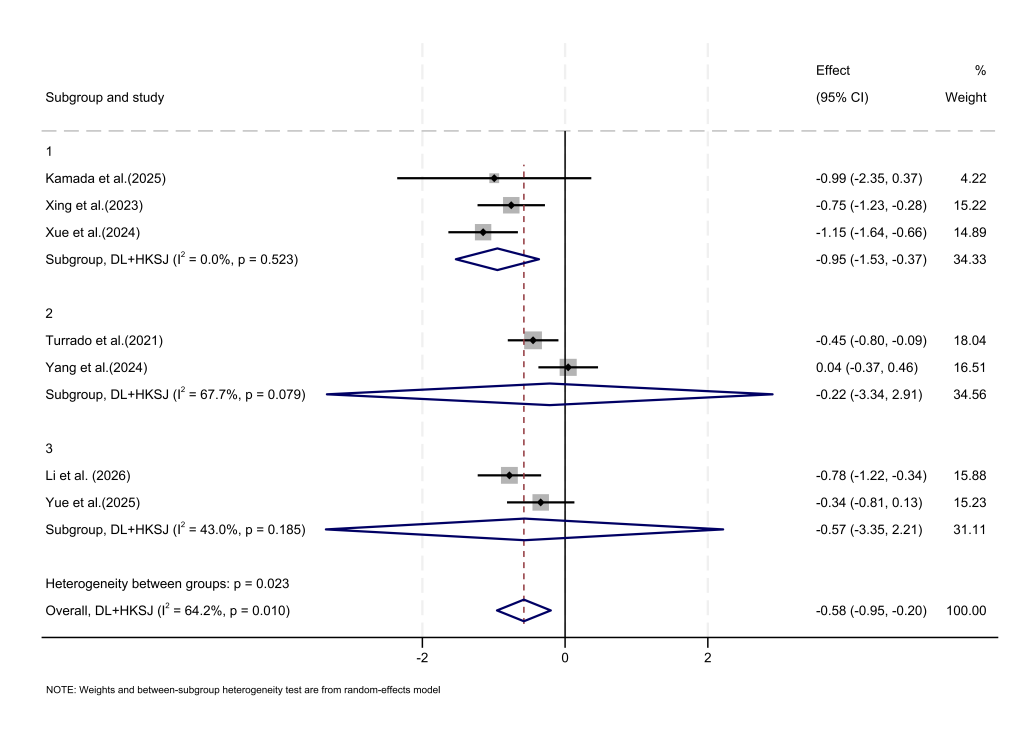
**

- - 1. **Duration**

**
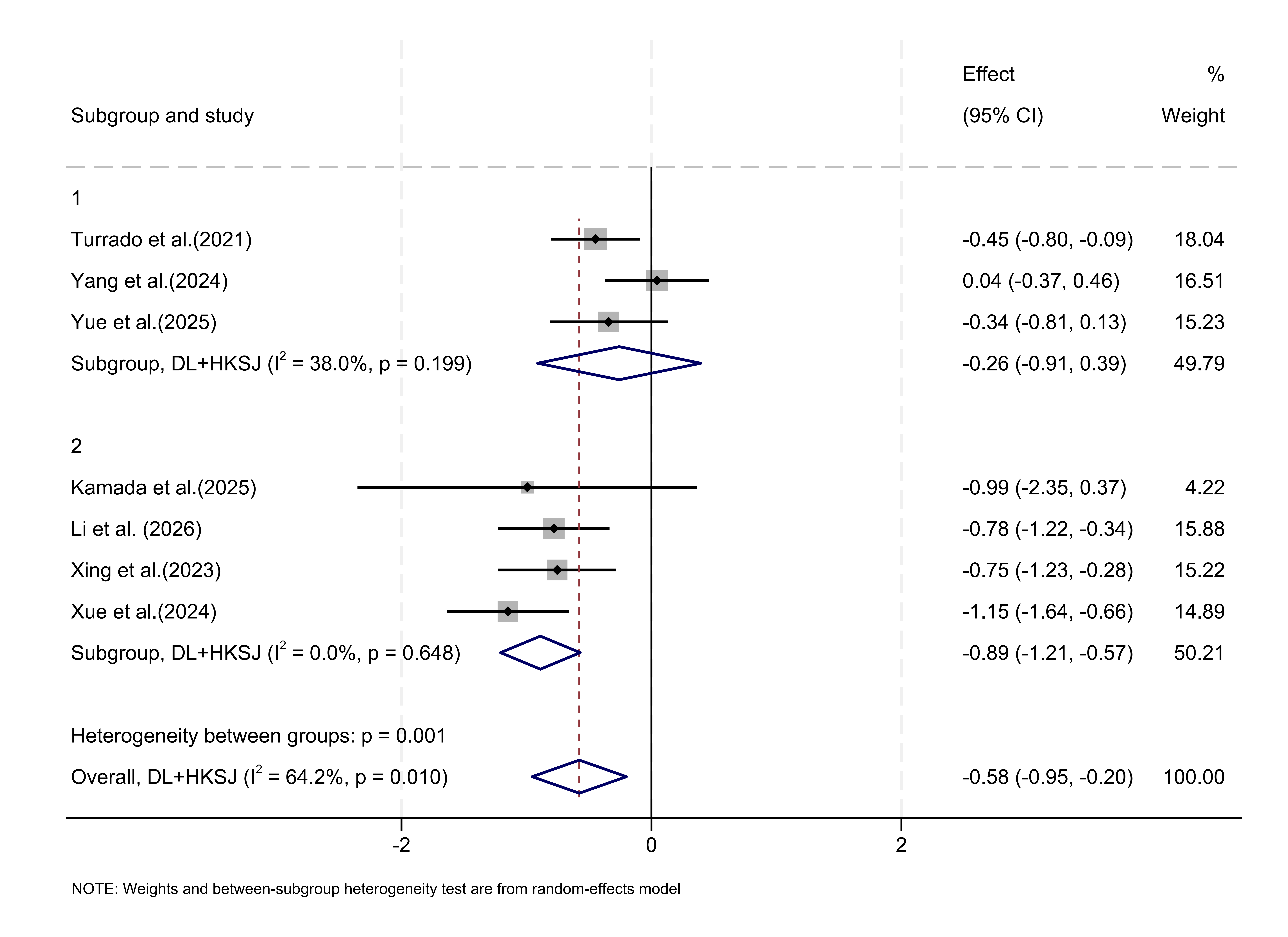
**

- - 1. **Number of interventions**


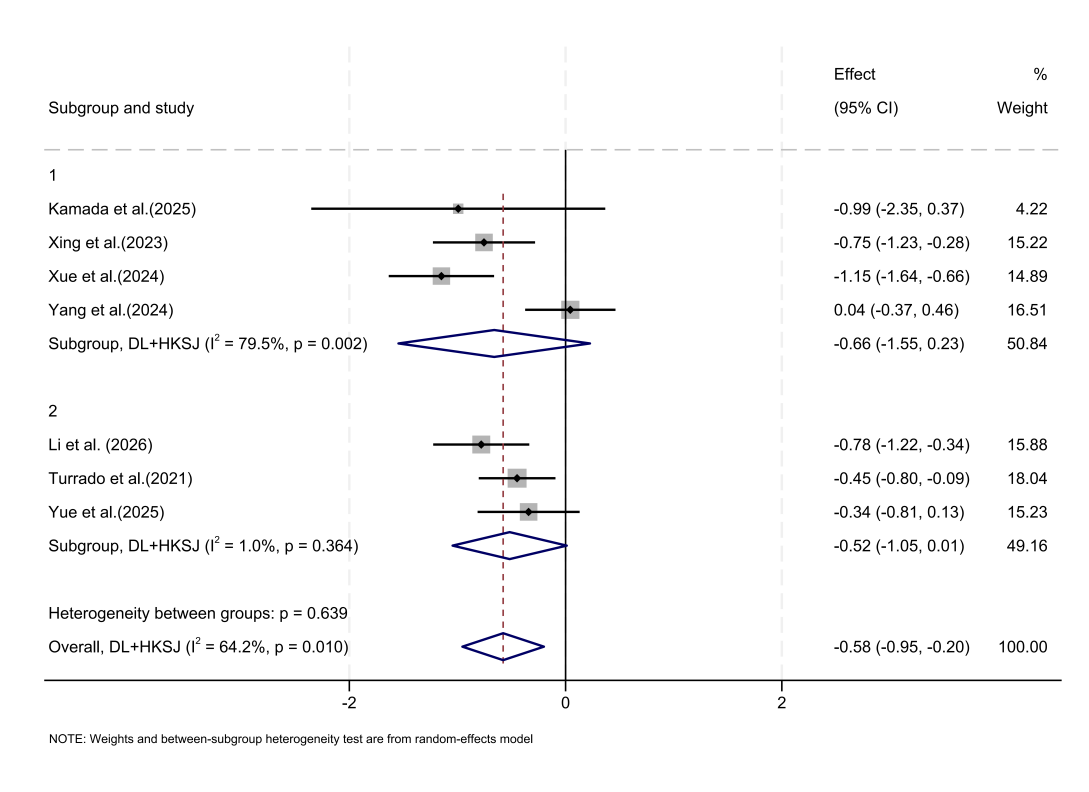


- 1. **Pain**
     1. **Time point**

**
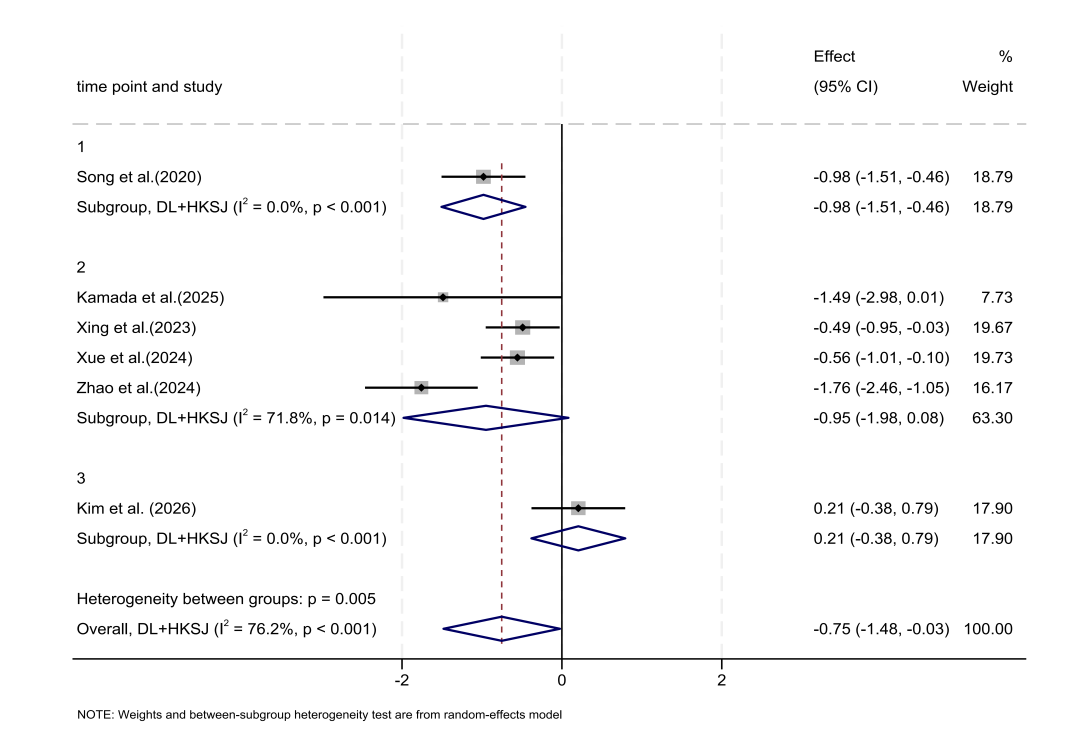
**

- - 1. **Setting**

**
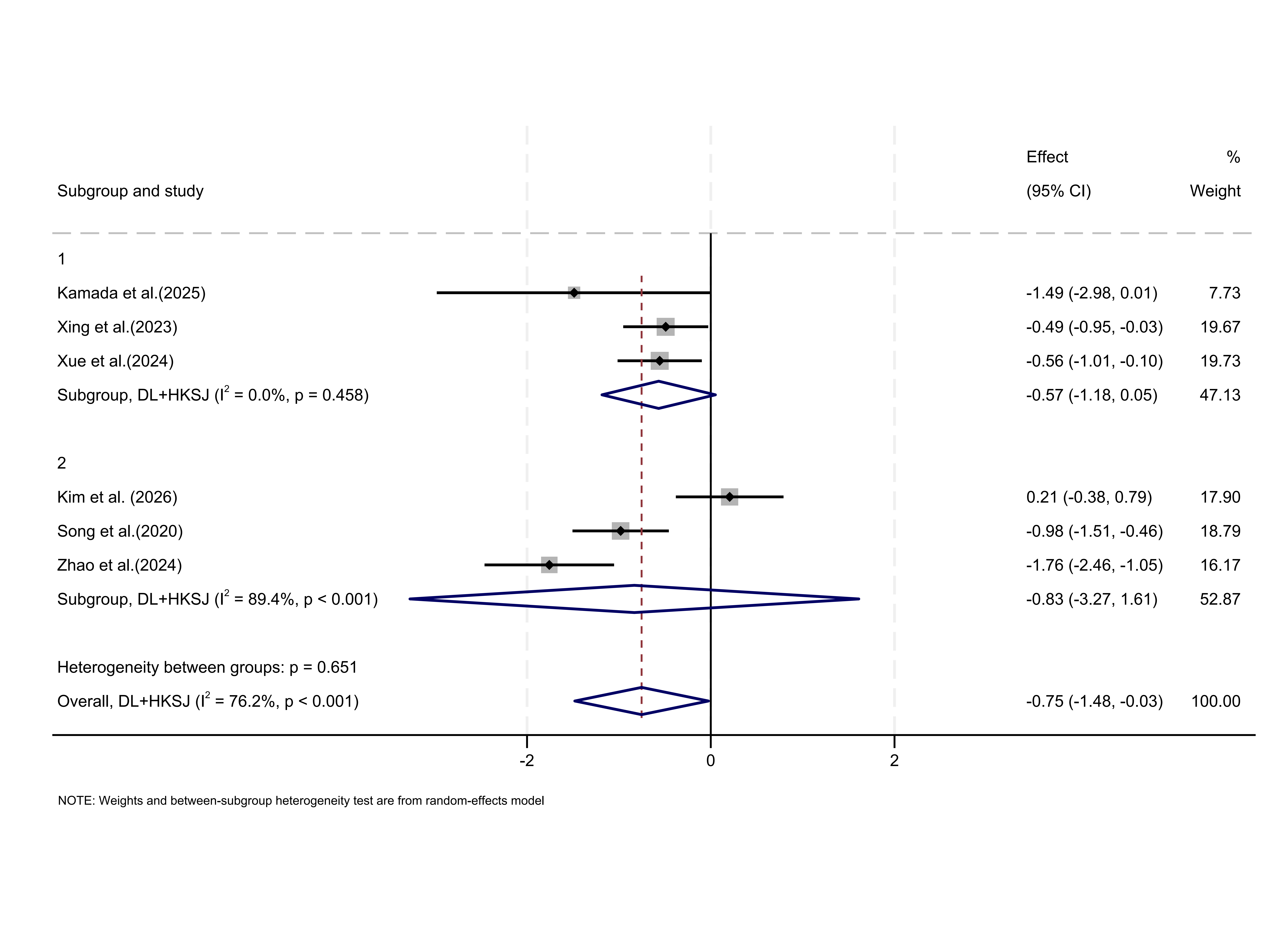
**

- - 1. **Duration**


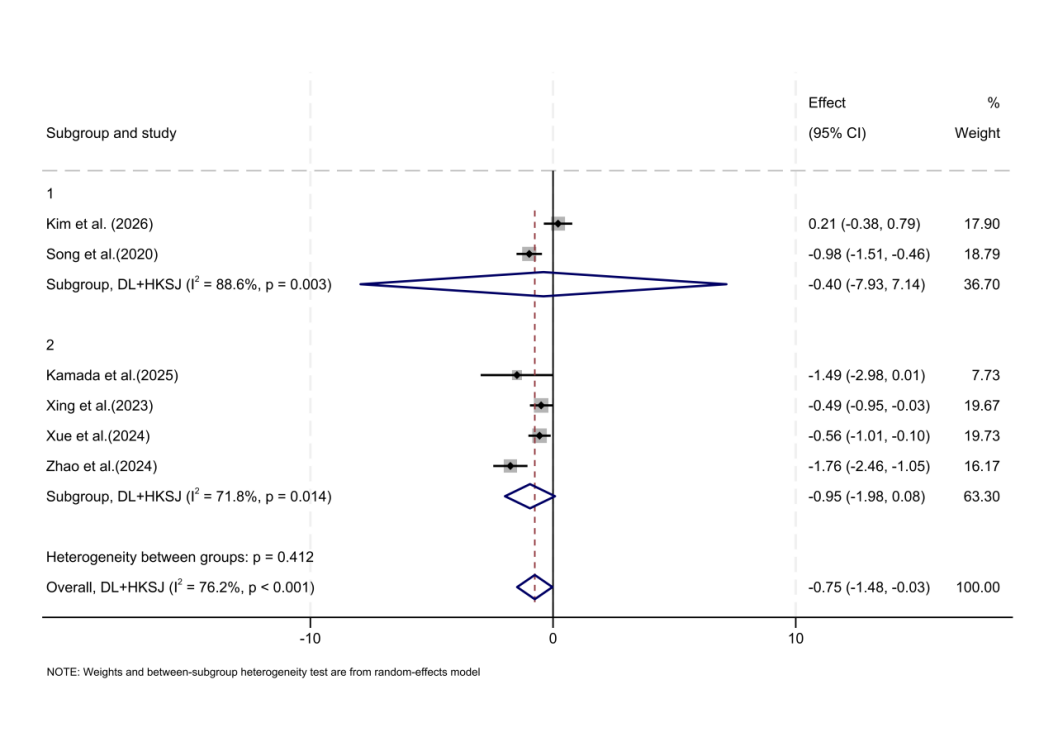


- - 1. **Number of interventions**


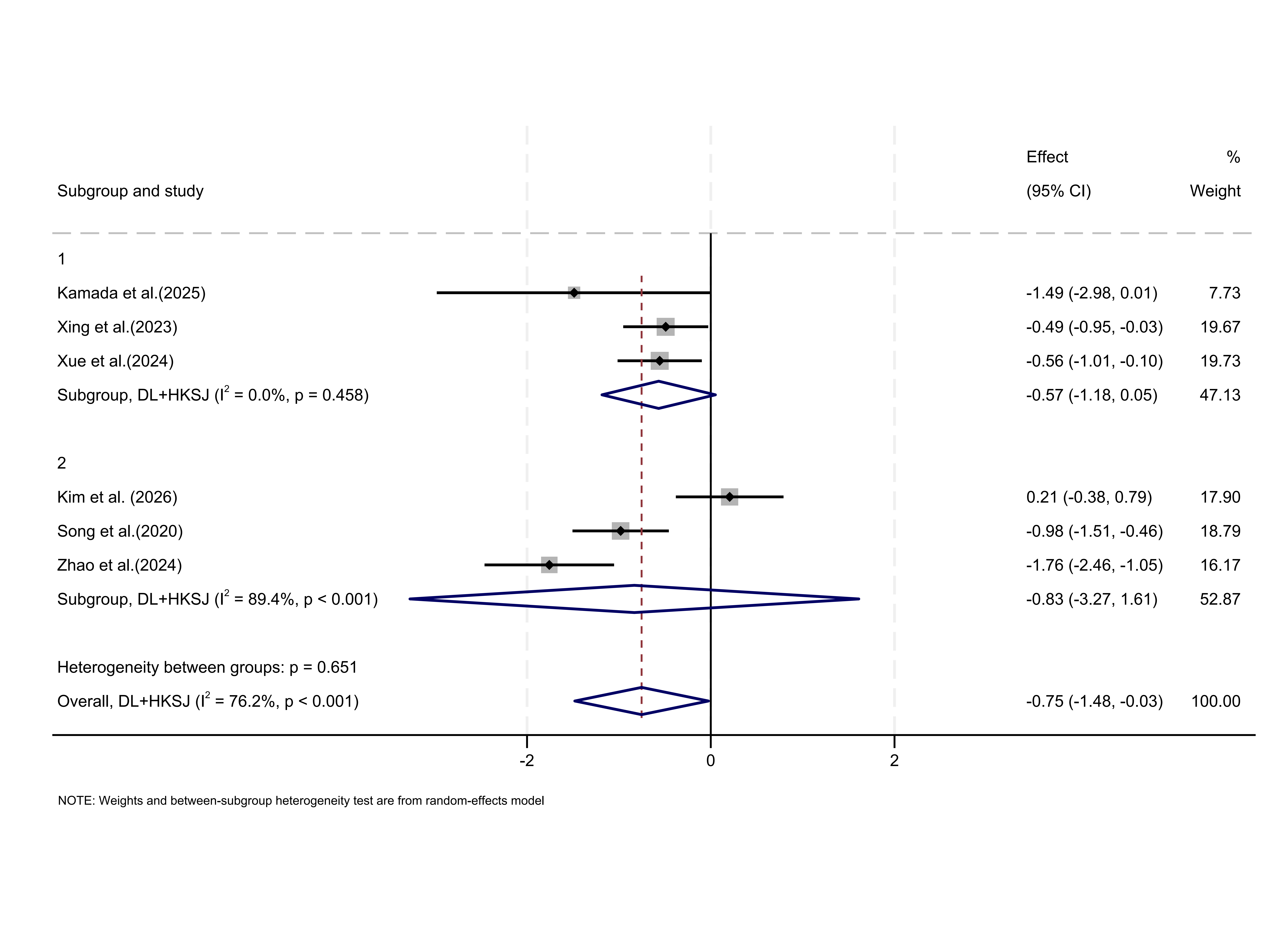


1. **Sensitivity analyses**
   1. **Anxiety**

- 1. **Pain**

- 1. **Length of stay**
